# The Last Universal Common Ancestor: emergence, constitution and genetic legacy of an elusive forerunner

**DOI:** 10.1186/1745-6150-3-29

**Published:** 2008-07-09

**Authors:** Nicolas Glansdorff, Ying Xu, Bernard Labedan

**Affiliations:** 1 JM Wiame Research Institute for Microbiology and Vrije Universiteit Brussel, 1 ave E. Gryzon, B-1070 Brussels, Belgium; 2 Institut de Génétique et Microbiologie, Université Paris Sud, CNRS UMR 8621, Bâtiment 400, 91405 Orsay Cedex, France

## Abstract

**Background:**

Since the reclassification of all life forms in three Domains (Archaea, Bacteria, Eukarya), the identity of their alleged forerunner (Last Universal Common Ancestor or LUCA) has been the subject of extensive controversies: progenote or already complex organism, prokaryote or protoeukaryote, thermophile or mesophile, product of a protracted progression from simple replicators to complex cells or born in the cradle of "catalytically closed" entities? We present a critical survey of the topic and suggest a scenario.

**Results:**

LUCA does not appear to have been a simple, primitive, hyperthermophilic prokaryote but rather a complex community of protoeukaryotes with a RNA genome, adapted to a broad range of moderate temperatures, genetically redundant, morphologically and metabolically diverse. LUCA's genetic redundancy predicts loss of paralogous gene copies in divergent lineages to be a significant source of phylogenetic anomalies, i.e. instances where a protein tree departs from the SSU-rRNA genealogy; consequently, horizontal gene transfer may not have the rampant character assumed by many. Examining membrane lipids suggest LUCA had *sn1,2 *ester fatty acid lipids from which Archaea emerged from the outset as thermophilic by "thermoreduction," with a new type of membrane, composed of *sn2,3 *ether isoprenoid lipids; this occurred without major enzymatic reconversion. Bacteria emerged by reductive evolution from LUCA and some lineages further acquired extreme thermophily by convergent evolution. This scenario is compatible with the hypothesis that the RNA to DNA transition resulted from different viral invasions as proposed by Forterre. Beyond the controversy opposing "replication first" to metabolism first", the predictive arguments of theories on "catalytic closure" or "compositional heredity" heavily weigh in favour of LUCA's ancestors having emerged as complex, self-replicating entities from which a genetic code arose under natural selection.

**Conclusion:**

Life was born complex and the LUCA displayed that heritage. It had the "body "of a mesophilic eukaryote well before maturing by endosymbiosis into an organism adapted to an atmosphere rich in oxygen. Abundant indications suggest reductive evolution of this complex and heterogeneous entity towards the "prokaryotic" Domains Archaea and Bacteria. The word "prokaryote" should be abandoned because epistemologically unsound.

**Reviewers:**

This article was reviewed by Anthony Poole, Patrick Forterre, and Nicolas Galtier.

## Background

Most biologists subscribe to Darwin's notion of an ancestor common to all living forms and so subscribe to its corollary, the existence of a Tree of Life [1]. Those who do not [2,3] may have exaggerated the occurrence of horizontal gene transfer by minimizing alternative interpretations, as discussed further. Since the ground-breaking discovery that every known living organism belongs to one of the three Domains, Bacteria, Archaea or Eukarya [4,5], the notion has given rise to the concept of Last Common Ancestor [6] or, according to Kyrpides et al. [7] and Lazcano and Forterre [8], of Last Universal Common Ancestor (LUCA), an acronym that combines the previous notion with that of the Universal Ancestor [9] and is sometimes used for Last Universal Cellular Ancestor [10]. There is however a wide variety of opinions regarding the cellular status (prokaryotic or not), homogeneity and complexity of this entity (the "community" concept [9]), depending on assumptions made on its mode of emergence, metabolic evolution and the nature of its genetic material. In particular, whether the progenote [4] -i.e. a primeval biological ancestor with a still rudimentary genotype-phenotype relationship and a RNA genome made of numerous minichomosomes – evolved into a LUCA still endowed with a RNA genome, or whether LUCA had already attained a later stage of evolution, with a RNA/DNA or DNA as genetic material, remains a matter of debate [[11-15] and below]. Moreover, to some authors, the LUCA is the direct ancestor of Bacteria and Archaea only, Eukarya being the product of some merging process between them [16-24]. Furthermore, recent developments concerning the origin of viruses and their possible role in evolution have opened new perspectives on the emergence and genetic legacy of LUCA [11,12].

The diversity of opinions concerning LUCA is in constant evolution and, new facts and ideas have been brought to attention in the last few years, These developments necessitate major adjustments in our approach; this is the subject of this paper. We focus on issues related to the cellular nature of the LUCA, its phylogenetic relationships, metabolic status, genetic redundancy and, last but not least, the question of how its predecessors emerged already complex. We certainly feel the need for a synthesis rather for than protracted polemics between entrenched visions. In our present state of knowledge, discussions about the origin of life and the status of LUCA remain largely theoretical; their value therefore is judged mainly by their explanatory power.

## Inadequacy of the term prokaryote

We have become used to deal with biological organization in the frame of a fundamental distinction between two types of organisms: the prokaryotes and the eukaryotes. This dual partition of the living world has lost much of its appeal with the discovery of the three Domains [5] and it has been proposed recently that the term "prokaryote" should be dropped altogether in favour of "microbes" [25]. Martin and Koonin [26] rightly pointed out the inadequacy of the term "microbes" and argued to maintain a "positive definition of prokaryotes" based on transcription-coupled RNA translation, (TT coupling) in keeping with the lack of a nucleus. However, this definition also is brought into question since the momentous discovery of a nucleus-like structure in some Planctomycetes, with a double membrane and pores [27]. Moreover, Poribacteria and some Archaea-like or ganisms also feature DNA-enclosing compartments [27]. It is not known whether TT coupling also applies in these organisms so that the validity of such a definition awaits confirmation. Nevertheless there are other reasons to relegate the term "prokaryote" to the historical record: (i) the prefix "pro" inappropriately suggests anteriority; (ii) this notion of anteriority of prokaryotes is usually associated with a very common prejudice in favour of an overall directionality in evolution, i.e. from the simple to the complex (a Lamarckian heritage) and, (iii) Bacteria branch off at the lowest position in a popular version of the tree of life [5]. As a result, it is often taken as a matter of course that LUCA must have been "something like a bacterium" and that many eukaryotic attributes evolved by gradual complexification, a notion whose inherent difficulties, however considerable, are often ignored [28-31] and has no compelling basis as a biological principle [32,33]. Indeed, what has increased in the course of evolution is order and its corollary, organization [34], complexity being a rather ill-defined and intuitive concept, except in the very abstract Rosennean definition of being "non-simulable", i.e. Turing incomputable, and in the objective, functional and molecular definitions referred to in the forelast section[35,36]. For example, a biofilm may be more complex than the simplest metazoans but is considerably less ordered. Organismal complexity, when it arises, is contingent on order.

What are the facts and the logic that we can use as a guide to discuss the nature of LUCA, its emergence and its legacy?

## Phylogeny of the three Domains. Proposed anciennity of protoeukaryotes

### Rooting the tree of life: what does it mean?

The rooting of the universal SSU-rRNA tree in the bacterial branch by Woese et al [5] rests on the phylogenetic analysis of paralogous proteins [37,38]; it has been seriously questioned. Forterre, Philippe and coworkers [10,39-41] and Caetano-Anolles [42,43] even suggested a root in the eukaryotic branch. This conclusion was rejected as a statistical artifact on the basis that eukaryotes cannot be older than prokaryotes since the former originated by endosymbiosis [22]. This rather trivial criticism however overlooks the notion that LUCA could have been a *protoeukaryote*, i.e. an ancestor to the cell line that captured the symbionts, an entity well distinct from Bacteria and Archaea. However, and most importantly the exact branching order has no bearing on the actual cellular architecture of the LUCA; a root in the eukaryotic branch would of course provide support for the notion of a protoeukaryotic LUCA but whichever would have branched off first, Bacteria [44] or Archaea [45], the LUCA could indeed have been a protoeukaryote announcing true Eukarya in many important respects, including critical properties of their membrane and cytoskeleton, and intron splicing.

### Reductive evolution

According to this view, explicitly advocated by a few authors [28,29,43,46-50], both Bacteria and Archaea are the product of reductive evolution, "streamlining", to revive a notion first presented in the wake of the discovery of introns [51,52]. As a matter of fact, it is usually not appreciated that there is **no evidence **that Bacteria and Archaea originated from anything that would deserve to be called a "prokaryote" in the current meaning of that word. It is interesting to note here the convergence between evolutionary thinking about animal phyla and lower organisms: just as a protoeukaryotic LUCA could be a rather complex but for ever lost intermediary state, and "prokaryotes" simplified evolutionary products, the Urbilateria (forerunners of bilateral animals) could have been vanished "elaborate ancestors" whereas flatworms and nematodes, once seen as ancestral because simple, are now regarded as "secondary simplified" or "degenerate" [33].

In line with the streamlining notion for the emergence of prokaryotes, and at a time the structure of the tree suggested that the earliest forms of life were extreme thermophilic Bacteria and Archaea, Forterre [46] proposed that the reductive evolution at the origin of Bacteria and Archaea had consisted in the "thermoreduction" of a non thermophilic LUCA. Before long however, doubts began to emerge on the thermophilic nature of the bacterial ancestor and Forterre [53] noted that certain bacterial features of thermoadaptation (particularly lipids) looked analogous, not homologous. More recent considerations on phylogeny and on the evolution of biological membranes indeed suggest that, contrary to Archaea, Bacteria emerged as non thermophilic descendants of the LUCA and that extreme thermophilic Bacteria arose by convergent evolution [[48] and below: **Origin of Thermophily and Biological Membranes]**.

### Introns already in the progenote?

Spliceosomal introns are found only in eukaryotes. Authors advocating eukaryogenesis by merging of Archaea and/or Bacteria consider that spliceosomal introns may have descended from type II introns present in one of the fusion partners [24,54]. However, to quote Delaye et al. [13], "there is no conclusive evidence that intron self-splicing and ribozyme-mediated RNA processing are truly primordial activities". Since the emergence of the complex spliceosome probably required a long period of time and since Collins and Penny [55] found evidence suggesting that an already complex spliceosome was present in the ancestor of all modern eukaryotes, it appears reasonable to infer its presence in a protoeukaryotic LUCA, from which most introns (and the spliceosomal machinery) would have been later lost in Archaea and Bacteria by reductive evolution. If the progenote genome was made of modules yet to be assembled into functional units, introns would have played an essential role in the formation of early genes by exon shuffling [[56] and references therein]; indeed, the gene-protein structure correlation postulated by the exon shuffling theory appears stronger in the subset of introns that are most likely to be ancient [56].

A splicing machinery would therefore have become essential very early and constitute an ancient feature of the LUCA that later might have facilitated coordination of transcription and export of transcripts from the nucleus [54]; the presence of a nuclear body in Planctomycetes and perhaps other "prokaryotes" [27] suggests that this evolution may already have been well advanced in the LUCA population. Moreover if the α-proteobacterium alleged to have been the precursor of mitochondria was endowed with an active enough intron splicing machinery to have impulsed a secondary wave of introns in a protoeukaryotic host, it could have been a microorganism perhaps already engaged in the streamlining process, but still closer to LUCA than its living descendants, so that both the mitochondrial precursor and its host would have used a spliceosomal machinery; as already stated above, the latter would have disappeared during reductive evolution of the protoeukaryote towards modern "prokaryotes", leaving them with the spare amount of introns actually found in Bacteria but also in Archaea (*Methanosarcina*). Actually, the fact that some introns have been found in Archaea weakens the argument proposing that eukaryotes emerged by fusion of a bacterium with an archaeon and that this very fusion sparked an intron invasion in an alleged intron-free archaeal host [24,54,57]. However such invasions would be expected to occur in a sexually outcrossing population [57,58] and some kind of primitive sexuality could have been a feature of a protoeukaryotic LUCA about to engulf the mitochondrial ancestor by phagocytosis [57,59].

### Eukaryotic gene promoters betray their progenotic origin

Eukaryotic gene promoters are essentially combinatorial structures. They consist in complex arrays of binding sites with defined specificities but susceptible of rearrangements without loss of function. This contrasts with their bacterial and archaeal counterparts that are very compact and not prone to rearrangements. We already suggested [[29] and references therein] that a eukaryotic-like promoter structure would be easier to conceive as ancestral than the converse because of its combinatorial potential for further evolution; moreover the imprecision, flexibility and redundancy of the genetic organization likely to have been inherited by LUCA from the progenote seems more in keeping with the complexity expected for a protoeukaryotic than for a prokaryotic promoter. Finally, during the streamlining process that gave rise to Archaea and Bacteria, another mechanism characteristic of these organisms may have evolved: the rRNA-mRNA interactions required for translation of polycistronic mRNAs.

We think therefore, that the combinatorial and variable structure of eukaryotic gene promoters suggests an ancestor with the type of loose, poorly organized genetic apparatus that would have been characteristic of the primitive and modular progenote, rather than the extremely compact and indeed "streamlined" organization of control and regulatory regions found in Bacteria and Archaea.

### How old is the nucleus?

If LUCA had a RNA genome and DNA synthesis was invented twice [11,60], we could consider the possibility that a membraneous compartment (let us call it a protonucleus) had already formed autogenously around the primeval RNA genome, so that the nucleus itself would not necessarily have emerged twice independently. Such compartmentation could very early have played a capital role in protecting RNA, in ensuring its correct partition at cell division and in separating replication and editing from protein synthesis. An interesting model [61] suggests that proteins of nuclear pores and coat vesicles (thus components of the endocytotic apparatus) could have been formed endogenously from defined protein structural modules. This model makes the emergence of the nucleus much less of a mystery than before and links nucleogenesis to the emergence of phagocytosis (see further). Moreover, as pointed out by P. Forterre in his comments on this paper, RNA "nuclei" still exist today since RNA viruses recruit host membranes elements to form compartments in which their replication apparatus is surrounded by one or two membrane layers with an opercule for communication with the cytoplasm [62].

This notion of endogenous nucleogenesis, here applied to a putative RNA protonucleus, contrasts with the hypothesis that the nucleus formed only once after engulfment of a crenarchaeal ancestor by a phagocytic "chronocyte", the RNA prekaryotic LUCA proposed by Hartman et al. [63,64]. In our view, the nucleus would have already appeared in a RNA LUCA and the RNA to DNA transition would have been the consequence of two independent viral invasions of such cells as suggested by Forterre to explain the differences found between the genes of DNA metabolism in Bacteria and Archaea/Eukarya [[11,12,44] and further].

The concept of a RNA LUCA has been criticized by Delaye et al. [13] who argued that (i) the monophyletic origin of ribonucleotide reductase (RNP) suggests a DNA LUCA; the evolutionary history of RNPs remains however unclear, making it difficult to establish the timing of the emergence of the different RNP classes [14,44,65]; (ii) fidelity of information transfer would have been an acute problem for a large RNA genome; Poole and Logan [14] however discussed evidence that high fidelity RNA replication and efficient proofreading could have been an attribute of a RNA LUCA. Though the jury is still out, the possibility of a RNA LUCA appears to have gained some ground in the last few years.

It seems that, on the whole, the model of a protoeukaryotic RNA LUCA is in keeping with current evidence. In particular, the antiquity of an already complex spliceosomal mechanism, appearing to have evolved before the last ancestor of living eukaryotes, is not easy to reconcile with eukaryogenesis by merging of prokaryotes. Rather, the LUCA itself may have been that ancestor, already endowed with the forerunner of the eukaryotic nucleus.

The occurrence of nucleus-like structures in some Planctomycetes, in Poribacteria (and perhaps some Archaea as well [27] is a striking feature that must be accounted for in evolutionary scenarios centered on LUCA. The possibility of early endogenous nucleogenesis by a rather straightforward mechanism [61] places the origin of the Planctomycete nucleus in a new perspective. The nuclear body of *Gemmata*, with its double membrane and its pores, is presently the closest approximation of a eukaryotic nucleus outside of its traditional Domain. There may be a relationship between this eukaryotic-like structure and the capacity for sterol biosynthesis, a feature of Planctomycetes [[66] and next section]. It is however not known whether these structures are really homologous nor is it known whether TT-coupling is the rule in this bacterial group; moreover, there are ribosomes in both the cytoplasm and in the *Gemmata *nuclear body. Is the *Gemmata *nucleus homologous and ancestral to the eukaryotic one or does it result from an independent event, indicating perhaps that nucleogenesis was not a rare and unique event? The fact that other Planctomycetes have less elaborate DNA compartments may either suggest that the process of compartmentation has stopped in these organisms short of completion, or indicate partial regression, leading in turn to suggest that the regression has been completed in most "prokaryotic lineages". These questions are presently unresolved but make Planctomycetes a fascinating subject for further investigations.

## Palaeochemistry and chronology of early eukaryote evolution

What independent indications are there that a eukaryotic cell line, still at the protoeukaryotic stage, could be ancient enough to qualify as LUCA? The chemical analysis of 2.5–2.7 billion years old Archaean sedimentary rocks (bitumens extracted from shales) found in the Pilbara region of Western Australia has pushed the possible existence of organisms possessing membrane containing sterols as far back as that era [67] since a complex distribution of steranes was discovered in those formations. At present, however, the indigenous nature of the sterol biomarkers is not fully established (see [68] for a discussion of the pros and cons) so that the relevance of these dramatic observations to the origin of eukaryotes remains uncertain. However, other chemical indications (carbon isotopic excursion of kerogens) indirectly point to the possible existence of protoeukaryotic organisms older than 2.5 billion years [67,69]. This isotopic pattern indeed suggests the occurrence of active methanogenesis, an archaean metabolism; if Archaea and eukaryotes share a common ancestor [5] – what in our view means if Archaea evolved by thermoreduction from the protoeukaryote (see further) – the latter must be older still. A recent analysis pushed the onset of methanogenesis as far back as 3.46 Gyr ago [70] but the interpretation of the data is contentious [71]. However, Chistoserdova et al. [72] demonstrated the presence of genes for C_1 _transfer reactions linked to methanopterin and methanofuran in Planctomycetes; their phylogenetic analysis suggests this pathway was very ancient (as much as 2.78 Gyr ago, [73,74]) possibly present in LUCA already. A favoured scenario suggests the genes remained in Planctomycetes, Proteobacteria and Euryarchaea but were lost in most known lineages [72].

Interestingly some Planctomycetes (*Gemmata*) were shown to produce *de novo *lanosterol and its isomer parkeol and to concentrate these substances in their double-membrane-bounded nuclear body [66]. This important observation places the interpretation of the diagnostic value of ancient steranes in a novel perspective since it suggests that Planctomycetes could have kept metabolic and morphological features of a protoeukaryotic LUCA. Alternatively, the capacity to synthesize sterols could have been acquired horizontally from a eukaryotic lineage [66]; the authors of this suggestion emphasize however that such a transfer must have occurred at a very early time. Indeed, two other but rather distant bacterial lineages, the Methylococcales and the Myxobacteria, also contain sterol biosynthetic genes [66]. Considering the great anciennity of this alleged horizontal gene transfer (HGT), it is not unreasonable to suggest that the donor may have been a protoeukaryote.

It is worth noting that Methylococcales and Myxobacteria share with the Planctomycetes a number of features that are atypical of most other Bacteria (intracellular membranes, unusual cell walls, complex reproductive strategies) and could indicate a relationship with a protoeukaryotic LUCA that would be closer than for any other living organism [27,72]. This is also supported by the exceptionally high degree of scattering found among functionally related genes in *Pirulella *(including a split rRNA operon) as compared to most prokaryotes [75]. Moreover, Planctomycetes and the related Verrucobacteria make proteins homologous to those of the eukaryotic cytoskeleton, such as integrin alpha-V, tubulin, actin and dynamin that could very well be of protoeukaryotic rather than prokaryotic origin [27,50,76]. It is still not clear whether the Planctomycetes, that were suggested to represent the most ancient branch of the Bacteria [77], really occupy this position [72,75,78,79] an issue that is notably difficult to resolve since most bacterial phyla branch off at a very deep level (see [27,50,80] and below, **Thermophily and the origin of bacterial membranes**).

However, even conflicting views on the exact branching order of the bacterial phyla still place the Planctomycetes among the deepest branches, along with the Poribacteria, another phylum of internally compartmentalized organisms [50,72,81]. No diagnostically eukaryotic fossils as old as the Pilbara bitumen [67] have been discovered, whereas contemporary or older bacteria-like fossilized objects have been reported; the first diagnostically identifiable Cyanobacteria are approximately 2.1 billion year old [82]. It is possible of course that the envelope of primeval eukaryotic precursors and other structures characteristic of eukaryotes were too fragile to have been preserved in metamorphosed archaean rocks [82]. Cavallier-Smith [83,84] doubted the possibility to recognize eukaryotic features in ancient fossils. This skepticism may be unfounded however since careful studies of 1800–1300 Ma fossils from Australian and Chinese formations, thus of a much greater anciennity than the date proposed by Cavallier-Smith for the emergence of eukaryotes (900 Ma ago), identify features strongly suggestive of eukaryotic morphology [82,85,86]. Moreover biomarkers extracted from a 1.64 billion-year-old old formation include steranes indicative of eukaryotes possessing advanced sterol biosynthesis [87].

## Eukaryogenesis by merging of prokaryotes or by phagocytosis?

### Models of fusion

The hypothesis of a protoeukaryotic LUCA ancestral to the bacterial and archaeal Domains starkly contrasts with the views [16-24] that regard the eukaryotic cell as the product of a fusion or merger between one or several Bacteria, or between Bacteria and an Archaeon. As noted by Caetano-Anolles [88], the "ring of life'[23] presented in support of an origin of eukaryotes by fusion of Bacteria, can be opened by assuming differential loss of genetic repertoires and give rise to a tree where Bacteria and Archaea appear as streamlined protoeukaryotes. However attractive the merging scenarios may appear at first sight from the metabolic point of view, or to account for the origin of the nucleus, they are not supported by available data [31,63,89-93]; the phylogenetic analysis of several proteins, including glycolytic enzymes, of components of the translation and transcription apparatuses and – more recently, of fold superfamilies [90]- indeed point to the existence of ancient eukaryote-specific proteins (ESP) and to a common ancestor for the three Domains. The fusion scenarios have little explanatory value in terms of genomic organization and for the origin of ESP's [31,94,95]. Furthermore, key proteins of the cytoskeleton previously considered to have been eukaryotic innovations were found to have homologues in Bacteria (particularly Planctomycetes and Verrucobacteria [27]) and Archaea [96]; they may have been inherited from a protoeukaryotic ancestor, perhaps much older than Bacteria and Archaea [97] rather than attest of a prokaryotic origin of the eukaryotic cytoskeleton. Similarly, the prokaryotic V4 domain has been presented as the likely ancestor of a key component of the eukaryotic vesicle transport system [98], whereas a protoeukaryotic origin could also explain the data. Likewise, the eukaryotic Ras superfamily was recently found to have homologues in Bacteria and Archaea; rather than suppose two independent prokaryotic origins followed by fusion between two such prokaryotes [99] one could assume their previous occurrence in the protoeukaryotic LUCA. Moreover, as discussed by Esser and Martin [100], current symbiotic models for the origin of eukaryotes by fusion of prokaryotic cells do not predict that genes from several bacterial groups (i.e. Spirochaetes and α-Proteobacteria) would contribute in a major way to the common ancestor of eukaryotes; however, such a multiple inheritance pattern is precisely what the hypothesis of a protoeukaryotic LUCA ancestral to "prokaryotes" would predict.

**Figure 1:**
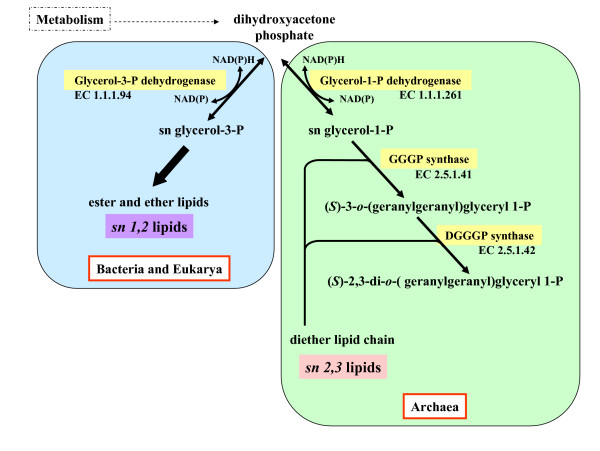
**Committing steps in the biosynthesis of membrane lipids**. The "primary divide" separating Archaea from Bacteria and Eukarya is outlined.

Furthermore, but for one instance of a γ-Proteobacterium living as endosymbiont within a β-Proteobacterium [101], there are no documented endosymbiotic associations between prokaryotes. It is actually surprising that the only mechanistic indication that bacteria might actually fuse or merge (the discovery of zygogenesis among Enterobacteriaceae by Gratia [[102,103] and ref therein] is not mentioned by proponents of fusion models. However, the mechanism is unknown, as is the actual extent of the phenomenon among bacterial groups; in our present state of knowledge, it certainly cannot account for the capture of an archaeon by a bacterium, whereas it might possibly explain the above-mentioned β-γ proteobacterial symbiosis [101]. When the fusion of an Archaeon with a Bacterium is considered [19,21,22,24] another difficulty is the incompatibility between glycerol membrane lipids of different chirality (*sn1,2 *fatty acid lipids and *sn2,3 *isoprenoid lipids as summarized in Fig. 1) [104] except if fusion actually involved engulfment, for which no precedent exists among prokaryotes; however, in that case, the preferential elimination of one type of membrane inside the host still requires an explanation. Besides, there are archaeon symbionts multiplying in eukaryotic cells [105,106] but no evidence was provided that they shed their isoprenoid membrane to replace it by a fatty acid one.

Wachtershauser [107,108] has proposed an ingenious alternative to maintain the idea of eukaryotes resulting from a merging process by assuming the latter took place between wall-less bacterial cells and "pre-cells" still at the LUCA stage, endowed with a racemic mixture of lipids (*sn1,2 *and *sn2,3*) but happening to be enriched by spontaneous molecular segregation in the bacterial type of lipids. This hypothesis rests on the possibility that LUCA would have been endowed for a significant period of time with a membrane made of a racemic mixture of lipids, in spite of their having a spontaneous tendency to segregate from each other, an eventuality that we find difficult to accept (see further **Thermophily and the origin of biological membranes**).

### A seducing hypothesis: Phagocytosis

Most importantly, the fusion scenarios referred to above underestimate the importance of phagocytosis; the hypothesis of engulfment of an α-proteobacterium ancestor in the capacity of mitochondrial precursor by a phagocytic protoeukaryote has lost none of its appeal, considering the well documented occurrence of prokaryotic endosymbiosis in eukaryotes [31,47,94,95,109,110]. Moreover, the most specifically articulated model of eukaryogenesis by symbiosis between prokaryotes [24] postulates a metabolic syntrophism based on hydrogen transfer between a *Myxobacterium *and an Archaeon as prerequisite for actual engulfment of the Archaeon by the Bacterium, followed by elimination of the archaeal membrane; this overlooks the fact that syntrophism based on H2 transfer between a Bacterium and an Archaeon, as it occurs in several microbial communities, does not need to be stabilized by endosymbiosis and subsequent gene transfer to the host genome in order to be efficient.

Endosymbiosis of methanogens within Protozoa -thus within phagocytic eukaryotes- do occur but the internalized Archaea conserve their individuality and, presumably, their isoprenoid membrane as well [106]. The mechanistically non-compelling nature of the endosymbiosis postulated by the proponents of this fusion model contrasts with the requirements for successful endosymbiosis of the mitochondrial ancestor; this seems to have been overlooked in criticisms of fusion models. Indeed, to explain the origin of mitochondria, we need actual engulfment of the future symbiont within an organism already endowed with a certain tolerance to O_2_, role postulated by de Duve for peroxisomes, [[94,95] and references therein].

Jekely [111,112] also discussed the implausibility of the models assuming fusion between prokaryotes from the point of view of membrane genesis and proposed that protoeukaryotes originated in microbial biofilms as "selfish cheaters" having evolved into predators that eventually became phagotrophs; mitochondrial symbiosis is seen as having triggered the formation of the nuclear compartment. In this model, the protoeukaryote originates from a true prokaryote having lost its rigid cell wall and developed endomembranes; in this respect it is reminiscent of Cavallier-Smith [83] hypothesis on the "neomuran" origin of eukaryotes. In spite of its prejudice in favour of a "prokaryote-to-eukaryote" transition, Jekely's interesting model [112] also emphasizes the importance of phagocytosis and proposes a plausible ecological context for the emergence of this property before the onset of endosymbiosis.

Phagocytosis thus remains an adequate mechanism to explain how the engulfment of future endosymbionts occurred. Hartman et al. [63,64], had already envisaged a phagocytic RNA ancestor (the "chronocyte") as precursors of eukaryotes; they proposed that the nucleus originated from the absorption by the chronocyte of an ancestor of the Crenarchaeotes with a DNA genome. This hypothesis however does not explain the specific elimination of the isoprenoid membrane from the engulfed Archaeon; it is also at variance with the well-documented model presented by Devos et al [61] to explain the endogenous origin of the nucleus. We nevertheless feel that elements of Hartman and Fedorov's scenario are particularly interesting to consider from two points of view:

(i) in Forterre's proposal [11,12] the RNA-DNA transition would have occurred in the viral world and an RNA-LUCA would have given rise to the three Domains as the result of invasion by different cell lines of the LUCA population by separate DNA viruses. This model, offered as an alternative to non homologous gene replacement, elegantly explains the disparities observed between the bacterial and archaeal/eukaryotic replication apparatuses. The former version of the model envisaged three transitions, one for each Domain, whereas recent *in silico *evidence suggests only two RNA-DNA transitions took place since the common ancestor to Archaea and Eukarya would appear to have possessed a DNA genome already [44].

Note however that a recent phylogenetic analysis of tRNA suggests the archaeal lineage may have been the most ancient one; it would have been followed by viruses, then by Eukarya and Bacteria [45]. Perhaps the evidence pointing to a DNA ancestor common to both Archaea and Eukarya [44] indicates that the same or very similar DNA viruses were responsible for the RNA-DNA transition in a precursor of Archaea and Eukarya. At any rate, Forterre's proposal on the primordial role of viruses in Domain genesis could concur with the hypothesis of a phagocytic RNA-LUCA but without adopting the idea of a nuclear origin by phagocytosis.

If the nucleus of *Gemmata *and the eukaryotic nucleus were homologous, it would at first sight be easier to conceive of a DNA- than a RNA-LUCA, unless, however, the different viral invasions involved in the emergence of the three Domains occurred in cell lines already endowed with an endogenous nuclear precursor, but still equipped with a RNA genome, as proposed above. If the conjecture of a phagocytic LUCA is correct, both Bacteria and Archaea would have lost phagocytosis. It is however conceivable that, in the LUCA community, phagocytic organisms would have coexisted with non-phagocytic cells [31] that might be the ancestors of what we call today "prokaryotes".

(ii) Hartman and Fedorov [63] insist on the selective value of a membrane able to build up a proton motive force which would have provided a determinant advantage to a phagocytic protoeukaryotic ancestor with a eukaryotic type of plasma membrane (perhaps an ancestor possessing peroxysomes already providing a certain protection against oxygen [94,95]). Now, modern eukaryotic membranes are not completely devoid of electron carriers and it is not known how the protoeukaryotic membrane would have been equipped in this respect, but there is little doubt that it was the development of electron-transport systems of the prokaryotic type that ultimately made efficient respiratory metabolism possible; therefore, the phylogenetic indications obtained by Castresana ([113], see below) that the last common ancestor already possessed proteins involved in respiratory electron transport could be interpreted at least in part in terms of a heterogeneous ancestral community [9,31,114] containing cells with plasma membranes already endowed with a certain respiratory capacity, rather than a metabolically "omnipotent" LUCA.

## Evolution of metabolism

### A globally heterotrophic, and microaerobic LUCA community

There are two basically different types of metabolism -autotrophic and heterotrophic- and, consequently, two opposite views for the origin of the first living cells; when the generation of metabolic energy is taken into account, these views are however not as irreconcilable as used to be considered [115]. At any rate, LUCA was not an immediate descendant of these primeval cells and its metabolic status is not expected to bear a direct relationship to their origin. In its present state, and whatever the exact branching order of the three Domains, the universal tree of life, with many deep-branching chemoorganotrophic types of microorganisms, is not adverse to the notion of a heterotrophic LUCA (or to the presence of heterotrophs in a LUCA community of diverse metabolic types). This contrasts with earlier emphasis on autotrophic (and hyperthermophilic) metabolism in alleged primeval cell lines at a time the tree of life featured many such organisms close to the root[116]. Actually, even if the primeval cells that preceded the LUCA had been autotrophic, evolutionary pressure from an environment containing organic substances, whether of living or still from prebiotic origin, would have promoted the advent of heterotrophic cells. A penetrating analysis of the phylogeny of gene families involved in energetic metabolism [113] further suggests that LUCA (or the LUCA community [9,114]) was endowed with a wide spectrum of bioenergetic capacities, including the paraphernalia of respiration and even a superoxide dismutase [91]); oxygen may thus have become an electron acceptor at a very early time, though its concentration would have remained very low (but perhaps already compatible with a micro- or nanoaerophilic metabolism [94,113]) before the massive increase progressively brought about by oxygenic photosynthesis and other processes [117]. It is usually assumed that oxygen is necessary for sterol biosynthesis (oxidation of squalene) but the possibility of an anaerobic pathway for squalene biosynthesis should be kept in mind [118]. Should this be the case, and despite indications for early participation of oxygen in metabolism, the protoeukaryote could have emerged in a totally anaerobic environment, perhaps still at the progenote stage.

### Gene content in a complex LUCA

A recent estimate of the minimal gene content of LUCA based on whole-genome phylogenies identified over 1000 gene families (between 1144 and 1529 when eukaryotes are included, [[91] and references therein]) with relatively low numbers (<150) of Archaea/Bacteria specific families. The results of this analysis contrasts with the notion of a "minimal" genome based on essential genes [119] and confirms the wide range of functional capabilities of LUCA, including all major aspects of cellular life. The study does not directly address the question of the root of the universal tree but Ouzounis et al. [91] nevertheless express their skepticism toward the notion of a bacteria-like LUCA.

## Thermophily and the origin of biological membranes

### Hyperthermophily and optimal temperature

Modern hyperthermophily appears to be an elaborated, acquired trait [13,120,121] and it is possible that LUCA, even if it were thermophilic, did not grow in the range of temperatures characteristic of modern thermophiles. In particular, the concept of "optimal temperature" (often quoted in the following lines because often referred to in the literature) may have sometimes misled people when considering adaptation to high temperatures [50]. *Pyrococcus furiosus *appears to be in a dire metabolic state at its so-called "optimal" temperature of about 100°C (H. Markl, personal communication cited in ref 50) and organisms such as *Thermotoga *struggle to compensate increased proton permeability in their "optimal" temperature range by an increased respiratory rate [122]. Therefore, the characteristics of modern hyperthermophiles probably reflect later adaptations to transient exposures and perhaps competition with other organisms but not an optimal niche, so that inferences made from modern hyperthermophiles on the thermal profile of organisms living in the era of LUCA may not be appropriate. The "optimum" effect would be essentially kinetic but not reflect physiological "comfort". Comparable observations have been made for adaptation to cold [123,124].

Moreover, according to Woese and Kandler [9,114], LUCA was a promiscuous community of organisms diversified to a certain extent in terms of physiology. As most organisms grow over a temperature range of 30 to 40 degrees, we suggest that the LUCA community was not a population adapted to a particular temperature range but would have consistently brought together cells with overlapping temperature domains, thus enlarging the basis for further evolution.

### Defining the thermal profile of ancestors

#### Phylogeny

The thermal profile of LUCA remains a matter of debate but the notion of a hyperthermophilic LUCA [116,125-128] is not anymore as firmly supported as it once appeared [29,48-50]. We will summarize the controversy and discuss whether a provisional consensus appears possible. The thermal regimes to be considered are, respectively: hyperthermophilic (optimal growth temperature (Topt) above 80°C, upper limit (Tmax) about 100°C), extreme-thermophilic (Topt 70°C, Tmax 80°C), and moderate-thermophilic (Topt 50°C, Tmax 70°C).

In contrast with earlier views presenting LUCA as hyperthermophilic, several recent phylogenetic analyses of the Bacterial Domain suggest that *Thermotogales *and *Aquificales*, originally thought to represent the earliest branching phyla in the bacterial Domain, may not occupy this position (see ref 79 for the opposite view). Analyses based on conserved rRNA sequences [77], large number of genes [129], DNA-dependent RNA polymerase (DdRp) [130], DdRp and other proteins [131], phylogenomic analyses of families of orthologous genes (Sculo, Lespinet and Labedan, unpublished) and other references in [49] gave phylogenetic trees where extreme thermophiles appear on secondary branches and members of the Planctomycetes/Verrucobacteria/Spirochaetes superclade cluster close to the root; the phylogeny of protein disulfide oxidoreductases [132] does not support a hyperthermophilic LUCA either. Ciccarelli et al. [133] proposed *Thermoanaerobacter*, a thermophilic Firmicute with a Topt of 75°C, as the earliest bacterial cell line; however this analysis diverges from the well established phylogeny of Archaea [134,135] and should therefore be regarded with caution. When reviewing the phylogeny of thermophiles, Lebedinsky et al [136] noted that some of the early diverged but as yet uncultured bacterial lineages seem to be mesophilic by their GC content or their habitat.

Because of the deep branching pattern of bacterial clades, it is particularly difficult to establish a firm phylogenetic profile of this Domain; it is therefore necessary to consider other arguments to understand the evolution of thermophily.

#### Compositional and biochemical data

Di Giulio [125,127,128] used a « thermophily index » (TI) based on the propensity of certain amino acids to be represented in thermophilic proteins, to correlate it with Topt, in order to predict the thermal profile of the ancestor of each Domain and of LUCA itself. LUCA was estimated to have been thermophilic or hyperthermophilic. Most of the data concern the signal recognition particle (SRP); they are widely scattered and the prediction was made [125] with a confidence interval of about 20°C; moderate thermophiles like some Bacilli are at the lower end of this range. Moreover, (i) correlations established for other proteins gave lower estimates with even wider intervals [125]; (ii) of the several estimates made with the SRP protein for the ancestor of Eukaryotes (none of whom grows above 62°C), most reach rather high values, that would be typical of moderate thermophiles or thermotolerant mesophiles [126-128]. It is therefore possible that thermal regimes were overestimated. This could occur if the correlation between the TI of SRP and Topt was biased towards higher temperatures.

Galtier et al. [137] predicted a mesophilic or at the most moderately thermophilic LUCA from a statistical analysis of rRNA base composition. These conclusions were criticized by Di Giulio [126] but nevertheless confirmed by Galtier in a later analysis taking into account site-specific variation (covarion model, Galtier, [138]). The temperature at which the earliest Bacteria arose was recently estimated from the melting temperature (Tm) of elongation factor (EF) proteins reconstructed from ancestral Bacteria [139]. The value fell between 64 and 73°C but was somewhat lower (61.4°C) when equilibrium frequencies in the amino acid replacement matrix were based on 31 protein families.

If we take the latter results at face value, they are compatible with a thermal regime comparable to that of several Bacilli but much less thermophilic than the hyperthermophiles *Thermotoga *or *Aquifex*, or even the extreme thermophile *Thermus*. Interestingly, it is precisely above this range of growth temperatures (Tm and Tmax between 60 and 70°C) that we find the various types of unclassical membrane lipids which suggest (see next section, [48,50]) that different lines of extreme thermophilic Bacteria emerged by convergent evolution from a non-extreme thermophilic ancestor(perhaps a moderate thermophile or a thermotolerant mesophile) ,, equipped with the classical *sn1,2 *fatty acid membrane lipids characteristic of most mesophilic Bacteria and all Eukarya.

The estimates based on EF Tm values call for some caveats however:

(i) Ancestral protein sequences were reconstructed across two alleged « competing bacterial phylogenies » in order to assess the effects of topology on the ancestral phenotype. The bacterial phylogenies considered are admittedly different but can hardly be considered as « competing » from the point of view of thermalprofiles since both place thermophilic bacteria at the root: either the extreme thermophile *Thermoanaerobacter *[133] or the hyperthermophile *Thermotoga*. [140]. Not surprisingly, the estimate is lower in the first case (64.8°C) than in the second one (73.3°C); the still lower estimate obtained from 31 protein families (61.4°C) was based on the first phylogeny. It would have been interesting to choose a really « competing » phylogeny among the several ones that place non-thermophilic Bacteria at the root of the bacterial Domain.

(ii) The authors stress the parallel between their estimate and the temperature of ancient oceans calculated from isotope ratios (δ^18^O and δ^30^Si); the validity of the approach has been questioned however, and temperate or even cold climates were argued for the early Earth [141].

(iii) The correlation between EF stability and Topt of *E. coli *and *Thermus *is good but it is not unusual to find a protein with a Tm higher than the Topt or even the Tmax of the host; we do not know whether the EF Tm-Topt correlation was as good during early cell evolution.

Taking these caveats into account, especially (i), it could turn out that the T derived from reconstructed EF proteins is an overestimate. On the other hand, 60 to 65°C, (a moderately thermophilic or thermotolerant range) would be at the lower end of Di Giulio'estimates and at the upper end of Galtier's estimates. Moreover, the value estimated by Gaucher et al. [139] concerns the emergence of ancient Bacteria; the average environmental T of LUCA could have been lower. Furthermore, as already stated above in the introductory remarks, the LUCA community could have been a mixture of populations with different temperature profiles; perhaps Bacteria arose among already moderately thermophilic or thermotolerant representatives of the LUCA community.

#### Conclusion about the thermal profile of LUCA

The data are in keeping with LUCA having been a moderate thermophile or a thermotolerant mesophile, perhaps a community with a broad temperature range, from which some descendants evolved towards extreme- or hyperthermophily by secondary adaptations. This provisional "consensus" is also compatible with the data reported in the next section.

### Defining the membrane lipids of ancestors

The emergence of the two main types of glycerol membrane lipids (*sn1,2 *fatty acid ester and *sn2,3 *isoprenoid ether as summarized in Fig. 1) is crucial to understand adaptation to high temperature and is directly linked to the origin of the Archaea ([29,48] and below). For the reduction of the keto group in dihydroxy acetone phosphate (DAHP), a glycerol 3 phosphate dehydrogenase (G3PDH) accounts for Bacteria and Eukarya, and a glycerol 1 phosphate dehydrogenase (G1PDH) for Archaea (Fig. 1). The two enzymes are not homologous but molecular modeling [142] suggested glycerol dehydrogenase (GDH), an enzyme found in some Archaea, some Bacteria and several Fungi, as ancestor of G1DPH (see also [143,144]).

From the phylogenetic point of view, the Archaea appear to have emerged as hyperthermophiles [134,135], though this type of inference is always by default [145]. The first Eukarya were probably mesophilic or at the most moderately thermophilic since no eukaryote living above 62°C (some fungi) has ever been identified. It could be that this limitation is a property of membranes consisting of a double layer of ester-fatty acid lipids, a feature eukaryotes share with mesophilic and psychrophilic Bacteria and would have been a feature of a non- or moderately thermophilic LUCA.

Indeed, whereas Archaea are characterized by ether-isoprenoids lipids which are suitable for life at high temperature and other stressing conditions [122,146] extreme thermophilic Bacteria that grow at temperatures above 65–70°C possess various types of membrane lipids that could represent convergent adaptations to life at high temperature (see early observations by Forterre [53] and [48,49]) whereas membranes of psychrophilic, mesophilic or moderate-thermophilic Bacteria appear to respond to different temperature ranges by variations in the degree of lipid saturation and the nature of side chains [122,147].

What is particularly striking however, is the fact that among membrane lipids of Bacteria presenting adaptations to various extreme conditions, either external (temperature, acidity) or internal (anammoxosomes sequestering hydrazine), numerous instances of di-glycerol ether lipids, or lipids containing one ester and one ether bond, even of transmembrane tetraether non isoprenoid lipids, have appeared [148-152]. These bacterial glycerol ether lipids however are consistently of the *sn1,2 *stereoconformation, in contrast to the *sn2,3 *conformation found, until now, exclusively among the archaeal isoprenoid membrane lipids (Fig. 1). Therefore whether ester or ether, a "primary divide" appears to exist between two kinds of cells: on one side the Archaea (*sn2,3*), on the other side the Bacteria and the Eukarya (*sn 1, 2*), as emphasized by Wachtershauser [104] and by the group of Koga [153,154].

There is general agreement that the combination of ether bond and isoprenoid side chains, in particular in the transmembrane tetraether configuration, ensures high stability and low proton permeability [122]. Archaeal lipids thus appear well suited for adaptation to extreme conditions such as high temperature, acidity and oxidation; their ether bond is also resistant to high alkalinity. It may therefore come as a surprise that psychrophilic Archaea, such as those discovered by DeLong et al [155] in the microplankton of cold oceans display the same type of lipid architecture, even in the tetraether configuration. The paradox is only apparent, however, because isoprenoid lipids can remain in a crystalline liquid state over a very broad range, from about 0°C to above 100°C [122]. It is therefore possible to understand how psychrophilic Archaea may have evolved from thermophilic ancestors (as their phylogeny suggests) while keeping the same type of lipids. In addition, Archaea respond to variations in temperature by variations in the composition of their membranes lipids (for example the proportion of caldarchaeol tetraether lipids increases with temperature in *Methanocaldococcus jannaschii*, [156] and lipid unsaturation increases at low temperature in *Methanococcoides burtonii *[157]).

By comparison with the clear-cut molecular adaptations to extreme conditions (such as temperature and acidity) displayed by archaeal lipids, it seems reasonable to attribute the same function (stability, low proton permeability) to bacterial lipids partially mimicking their archaeal counterparts, such as lipids with ether bonds, sometimes in the tetraether configuration (see references above). The tetra-ester lipids and the long-chain diols found in some thermophilic Bacteria [158,159] may also contribute to membrane stability. The complex glycolipids found in *Thermus *and related organisms, display bulky head groups that presumably enhance stability [160]; they were found to increase in proportion with the growth temperature [152,161,162]. Note however that these are not isoprenoid lipids, which may explain, at least in part, why none of the so-called hyperthermophilic Bacteria can grow in the same temperature ranges as hyperthermophilic Archaea.

By contrast with the monotonic composition of archaeal lipids, the high variability observed among Bacteria adapted to extreme conditions (high temperature, acidity, hydrazine sequestering in anammoxosomes) but belonging to different branches of the Domain thus suggests that these adaptations are the result of evolutionary convergence from a non extreme thermophilic LUCA with membrane lipids in the *sn1,2 *configuration, presumably fatty acid lipids linked to glycerol by ester bonds. By contrast, the Archaea would have emerged as thermophiles from the start by inventing the *sn2,3 *isoprenoid ether configuration. We therefore suggested [29,48] that Archaea would have emerged by "thermoreduction" – to use Forterre's terminology – from a non thermophilic LUCA under strong selective pressure for adaptation to high temperature; on the contrary, in contrast with the original thermoreduction hypothesis [46], formulated when the tree of life still suggested that early "prokaryotes" were all extreme- or hyperthermophiles, Bacteria would have been originally non-thermophilic, having emerged by reductive evolution from the LUCA as a separate group at another time than Archaea; the different bacterial lipids resulting from convergent evolution would have kept the original *sn1,2 *configuration.

### The occurrence of the divide between sn1,2 (ester/ether-fatty acid) and sn2,3 ether-isoprenoid lipids

A major question is how this major divide occurred (Fig. 1). We consider as unlikely that *sn1,2 *fatty acid and *sn2,3 *isoprenoid membrane lipids independently replaced the mineral membranes of a non cellular but compartmentalized ancestor to create the two groups of prokaryotes. Indeed, arguing from the non homology of the membranes of Archaea and Bacteria in favour of a non cellular ancestor and the independent emergence of the two types of lipids [22,153,163] overlooks the fact that all organisms can synthesize both fatty acids and isoprenoids, and that, at least in theory, it is possible to retrodict both pathways to an ancestral mode of lipid synthesis [104].

For Wachtershauser [107], heterochiral membranes, with the two different types of lipids, preceded the *sn *divide for a long period of time; the first lipid were synthesized as a racemate, perhaps non enzymatically, the first enzymes to catalyze their formation were non-enantiospecific and later replaced by specific ones. Pereto et al. [143] favoured a similar scenario. Such membranes should be unstable however [164]; the mixed membrane therefore would have spontaneously segregated the two different types of lipids, creating organisms with either *sn1,2 *or *sn2,3 *(i.e., in Pereto et al. [143] Bacteria and Archaea, that would later have merged to generate the ancestor of eukaryotes).

We see several difficulties with this scenario. The idea of a non-enantiospecific enzyme is not very much in keeping with our current appreciation of enzymatic specificity. There is well a CDP-archaeol synthase that does not recognize ester or ether bonds between glycerol and hydrocarbon chains nor the stereostructure of glycerophosphate but mainly the geranylgeranylchains [165]; however the notion that the first step in the synthesis of isoprenoid ether glycerol lipids and all downstream enzymes would have been non-enantiospecific [107] looks a very constraining hypothesis. Moreover, the very instability of heterochiral membranes that underlies the idea of spontaneous segregation, might be so great (as suggested by the behaviour of racemic mixtures of D- and L-myristoyl-alanine: a strong chiral dicrimination in a few minutes, followed by chiral segregation into D- and L- domains in about one hour [166]) that the persistence of such membranes over a significant period of time appears problematic. Moreover, the phylogenetic inference that the subunits of protein translocase, which operate in a lipid environment, appears to have been present in the ancestors of Bacteria and Archaea suggests stable lipid membranes [108,167]; in the absence of experimental evidence, it seems questionable that such membranes could have been heterochiral. Besides, the emergence of the two enantiomeric membrane lipids is presented almost *in abstracto *as a spontaneous symmetry breaking process, without any attempt to relate it to environmental conditions that may have presided over the emergence of Bacteria and Archaea. Wachtershauser [107] rejected the idea that membranes with *sn2,3 *lipids emerged from an ancestor equipped with *sn1,2 *lipids as "counterselective ", arguing that an organism that would be in the process of such a reconversion would be too unstable, even if, on the other hand, he paradoxically assumes that heterochiral membranes could have persisted for several hundred million years. To fully understand the controversy, it must be recalled that Wachtershauser favours an origin of life at high temperature and subsequent evolution from a hyperthermophilic universal ancestor toward mesophilic and psychrophilic descendants, going as far as claiming that the reverse is "impossible" [168] (see however [50] for a discussion proposing mechanisms for progressive adaptation to thermal tolerance and thermophily; see also a recent report giving a striking example of adaptation to thermal tolerance by multiple symbiosis [169]) Wachtershauser therefore has no incentive to suppose that the emergence and segregation of organisms having acquired the capacity to synthesize *sn2,3 *isoprenoid lipids could have occurred under strong selective pressure for adaptation to high temperature in a mesophilic organism containing only *sn1,2 *fatty acid lipids, *which is exactly what we proposed *[29,48] and placed the putative reconversion process in a totally different perspective. Moreover, the fact that archaeal lipids are *sn2,3 ether *and that many extreme thermophilic bacteria also have *ether *lipids argues for the emergence of this type of lipid under selective pressure.

However our previous proposal [29,48] presented the transition *sn*1,2 fatty acid -*sn*2,3 isoprenoids lipids without specifying putative steps; as it is unlikely that the shift in stereoconfiguration and in the choice of side chains were simultaneous, we feel the necessity to be more explicit. Perhaps the membrane of the precursor of Archaea was first selected to contain isoprenoid ester lipids (providing already some adaptation to adverse conditions such as high temperature), then gave rise to isoprenoid-ether lipids (under selection for further adaptation) and this step may have automatically favoured the selection of the *sn*2,3 configuration. Indeed, GGGPS (the enzyme catalyzing the formation of the isoprenoid-ether bond, fig 1) displays a strong preference for G1P with respect to G3P [144]; if this is an intrinsic property of all GGGPS (or if a G1P-inclined GGGPS was the only one around at the time of selection of the lineage that was to persist), it is conceivable that the selection or the recruitment of a G1PDH followed suit. In the meantime some *sn*1,2 glycerol isoprenoid ether lipids could have been produced, to disappear later on with the recruitment of G1PDH as a enzyme providing a more adequate substrate. In this respect it is interesting that G1PDH is not an archaeal exclusivity; in fact both G1PDH [143] and GGGPS [144] have been found in several Bacteria where their present function is unknown. Both enzymes could have been present in the LUCA community; alternatively, under strong selective pressure, G1PDH could have been "borrowed" by HGT from a bacterium or recruited as a novel enzyme from a GDH. The next enzyme that completes the formation of a C20–C20 diether lipid (digeranylgeranylglyceryl phosphate synthase (DGGGPS), see Fig. 1) could have been recruited from the family of prenyltransferases that contains several membrane-intrinsic proteins in all three Domains [170]. Of course, in this view, the so-called "primary divide" between *sn*1,2 and *sn*2,3 glycerol lipids now appears as secondary!

Payandeh and Pai [171] showed that GGGPS probably originates from duplication and fusion of an ancestral gene coding for a (β-α)_4 _half-barrel. In their "lipid capture model" they proposed that GGGPS appeared in a bacterial-like ancestor, paving the way to the formation of an isoprenoid-lipid membrane (after recruitment of a G1PDH and a DGGGPS), by automatic segregation from a transient heterochiral membrane. The model has the advantage of not postulating a protracted heterochiral state as an intermediate, in keeping with our earlier proposal [48], but no assumption was made regarding the selective pressure that could have influenced the process. Moreover the authors favour eukaryogenesis by fusion between an Archaeon and a Bacterium, perhaps because if they assumed a common ancestor for all three Domains, their hypothesis would imply that Archaea and Bacteria diverged from a line already distinct from LUCA; this would not be in keeping with the well documented view that Archaea and Eukarya share an ancestor distinct from that of Bacteria.

We want to mention in passing that other lipids could have preceded glycerol lipids, perhaps at a very early time; Wachtershauser ([104], see also [48]) suggested that sphingolipids could have been the primeval ones. Sphingolipids are today represented in a few Bacteria, absent from Archaea (where they could have disappeared when the *sn*2,3 glycerol lipids became dominant) and ubiquitous in Eukarya.

To conclude, we definitely prefer a Darwinian working hypothesis to the fortuitous emergence of enantiospecific enzymes followed by automatic segregation of two types of lipids. Since all organisms appear to be capable of synthesizing both fatty acids and isoprenoids, our scenario suggests that no major, improbable reconversions would have had to occur when converting one type of membrane into the other one. Genetic experiments on the degree of flexibility of GGGPS stereospecificity could provide interesting results. Useful information would also be obtained from genetically engineered organisms able to synthesize both types of lipids, if these turned out to be viable; perhaps they would if they were engineered so as to conditionally synthesize the two types of lipids.

## LUCA was genetically redundant; differential loss of paralogues created numerous phylogenetic discrepancies

In the above we repeatedly stressed that the LUCA does not appear to have been a simple, minimal system from which everything eukaryotic emerged by further complexification. In particular, phylogenetic inferences on its metabolism and gene content give a sophisticated picture [29,43,91,114,172,173] that can in part be understood in terms of a diversified and promiscuous community, but also taken as a sign of generalized genetic redundancy. It is indeed very likely that most cells in an ancestral community having engendered the diversity of metabolic functions found in the three Domains possessed more than a single copy of every essential gene as well as numerous paralogous genes. This redundancy could have been selected for as an important survival factor for cells with a still primitive, not fail-safe division mechanism. An important consequence of both this redundant genetic inventory, and of the complexity of the communal LUCA population, is that its descendants, in any one of the three Domains, will have inherited only one of many of the genes that were present in more than one exemplar in the ancestral pool. A striking example of this is the phylogenetic analysis of carbamoyltransferases by Labedan and colleagues [172,173]; the intricate topology of the distribution of both aspartate- and ornithine-carbamoyltransferases (ATCase and OTCase, respectively) among the three Domains could readily be understood by haphazard loss of gene copies in different lines of descent when it was recognized that any ATCase or OTCase belongs to one of two families that can be traced back to gene duplications having occurred most probably before emergence of the LUCA (Fig 3 of [172]). The statistical validity of these very ancient paralogies was confirmed by the unfailing correspondence found between the type of ATCase gene inherited and the structural class of the corresponding enzyme [173]. Moreover, a tree made with nearly 3,000 homologous carbamoyltransferases corresponding to at least five different enzymatic activities (our very recent unpublished results) confirms that the primary duplications that produced differentiated genes from an ancestral, substrate ambiguous carbamoyltransferase was already an ancient event in the evolution of LUCA. Most probably, these primeval enzymes present in the ancestors of LUCA were already endowed with a surprising functional diversity. Such a specific protein history is unlikely to be an isolated case as studies on other enzymes suggest [39,93,174-177]. Moreover, paralogies may go unnoticed if gene duplicates remain cryptic as the result of inactivating mutations [178].

Therefore, when attempting to build a phylogenetic tree with genes encoding proteins, many unpredictable discrepancies with respect to the classical SSU-rRNA tree are *expected *to turn out because of loss of paralogues. Numerous such discrepancies have indeed been observed but, in most cases, attributed to HGT without other justification than the occurrence of the discrepancy itself. Several authors have pointed out that such a systematic bias is abusive and that successful HGT, especially between phylogenetically distant organisms, has to go through several steps, none of which appears particularly likely, even when ecological proximity is granted [29,49,89,179-182]. In contrast with the transfer of genes between related organisms or between members of a group such as the Proteobacteria, where arguments independent from statistics support the occurrence of HGT (presence of transposons, integrons, genomic islands, presence or absence of whole sets of genes in different strains from the same species, especially pathogens), acquisition of a foreign gene from a phylogenetically distant organism to complement a defective mutant is less likely than replacement by an intact exemplar from cells of the same species; moreover successful HGT requires replication, maintenance and efficient expression of the transferred gene, which, in the case of interdomain transfer compounds difficulties. Furthermore, inferring the incidence of interdomain transfer from the apparent frequency of foreign but nevertheless mostly bacterial-like genes hosted by *E. coli *[2] is misleading [29]. In fact, a rigorous phylogenetic analysis confirmed that most genes appear to be vertically inherited but suggested that metabolic genes (that may confer direct physiological advantages) could be more prone to HGT [183]. It is not the place here to again discuss these arguments in detail (see references above) only to stress the point that the loss of paralogous gene copies in descendants of LUCA is not just an alternative explanation for phylogenetic discrepancies, it is an actual *prediction*.

In addition, it is possible that certain phylogenetic discrepancies are due to differential loss of paralogues created just ahead of a bifurcation leading to a phylogenetic anomaly; this might also explain a large number of events attributed to HGT; various chromosomal rearrangements increasing gene copy number indeed occur in bacteria, with variable frequencies [184-186].

In many cases it would be difficult to distinguish between HGT and gene loss; however, in the very formulation of alleged HGT patterns, it is sometimes apparent how close that interpretation comes close to the unmentioned alternative; see for example [144] when Boucher et al. conclude from their phylogenetic analysis of genes involved in isoprenoid synthesis, that some of the postulated HGTs must have taken place "prior to the diversification of these groups" (refers to "particular orders of Archaea") or represent transfer from eukaryotes to Archaea. The bias for HGT even takes the form of circular reasoning in a review stating that "the fixation and long-term persistence of horizontally transferred genes *suggests *(our emphasis) that they confer a selective advantage on the recipient organism" ([187], page 709). We conclude that the intrinsic likeliness of differential gene loss and the unlikeliness of HGT between organisms as different and distant as Archaea and Bacteria or even between many phyla within the same Domain (requiring several events without any obvious selection in most cases) bring in doubt the rampant character attributed to HGT by many authors. It could very well be that loss of paralogues (from LUCA and created by later duplications) accounts for a large proportion of events attributed to HGT, especially between Domains (an event that would compound difficulties). It should be clear that we are not rejecting the notion that a certain proportion of phylogenetic incongruencies are due to HGT; even between Domains, the transfer of genes with pleiotropic effects (such as reverse gyrase or other topoisomerases [44,188] appears to have occurred, presumably under selection; this type of event seems however infrequent and contrasts with the indiscriminate recourse to HGT found in many publications.

It may again be emphasized that this view is in keeping with a shift in our appreciation of the nature of LUCA. It is not anymore taken for granted that LUCA was a "simple or primitive cellular entity" [189]; moreover the emergence of the branches leading to the two "prokaryotic" Domains also is likely to result from a complex process involving constant and mutual genomic additions to the evolving cells, until the moment the cellular subsystems we know as Domains "crystallized" [9] as organismal lineages, becoming by and large refractory to further genetic exchanges except perhaps under strong selective pressure. Consequently, without further evidence for the actual occurrence of HGT and its real scope, it appears premature to challenge the existence of a Tree of Life [2,3].

## The evolutionary position of the LUCA

### A communal RNA LUCA

**Figure 2:**
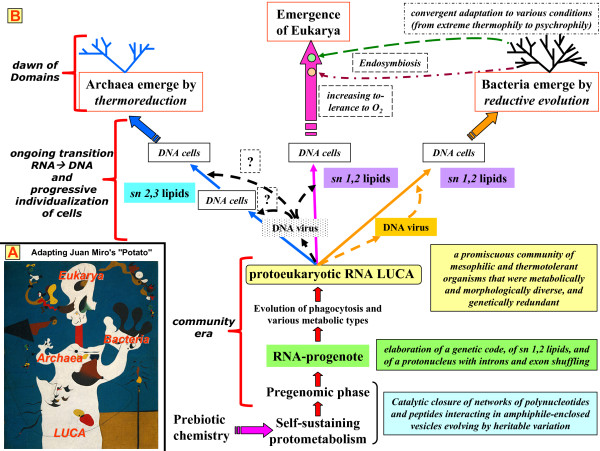
**Birth and legacy of the Last Universal Common Ancestor (LUCA)**. A large, evolving and promiscuous community stretches in time from the origins to the immediate precursors of the three Domains (and perhaps of many other ones, presumably abortive). (A) The "sprouting tuber" analogy [49], illustrated by Juan Miro's "Potato" [Copyright: The Metropolitan Museum of Art, New York, New York, USA. The Potato (1928) by Joan Miró (Spanish, 1893–1983). Oil on canvas; 39 3/4 × 32 1/8 in. (101 × 81.6 cm). Jacques and Natasha Gelman Collection, 1998 (1999.363.50). ^© ^2000 Artists Rights Society (ARS), New York/ADAGP, Paris]; (B) Progression from the inorganic to self-replicating entities via a qualitative jump to complexity by catalytic closure, and further to cells with a DNA genome. The diagram illustrates the proposition that viruses originate from a cellular precursor [45] and that viruses are responsible for the RNA-DNA transition in Bacteria on one side and Archaea/Eukarya on the other [11,12,44]. The exact branching order is not specified (see [44,45] and text). See text for details of the sn1,2→sn2,3 lipids transition. The onset and course of the reductive evolution leading to Archaea or Bacteria are not indicated in detail. We conceive of this process as having occurred in several steps, more a succession of evolutionary crises than a gradual transformation; it involved the emergence of cells with membranes fully competent in electron-transport-driven energy harnessing and the RNA-DNA transition.

Before examining the origin of a complex LUCA in the last part of this paper, we will summarize our position regarding the cellular constitution and the immediate legacy of the LUCA (see Fig. 2). An important point is that the "primary divide" in the emergence and evolution of biological membranes is compatible with the scenario of a major divergence from a multiphenotypic RNA LUCA community into Bacteria and Archaea/Eukarya.

We have discussed arguments that make the notion of a RNA (or possibly a RNA/DNA) LUCA less improbable that it appeared only a few years ago. A DNA LUCA is not excluded (see [13] for a vigorous defense of the concept, also [107] though with very different arguments), but recent developments support the hypothesis of a RNA LUCA that would have evolved into descendants with DNA as genetic material by processes implying the intervention of viruses [11,12,44]. The idea that such a RNA LUCA was a phagocytic cell, (or at least belonged to a community containing such organisms) has also come on the foreground [31,63]. We considered the possibility that a *bona fide *DNA nucleus could have emerged in a RNA LUCA containing a protonucleus enclosing the RNA genome, in order to minimize the number of independent events to be postulated. Further research on the occurrence of comparable structures throughout Bacteria and Archaea, on the phylogeny of their components and mechanisms of information transfer, could have a major impact on our perception of LUCA.

For reasons we hope to have made clear all along this discussion, we will not come back on the models of eukaryogenesis by merging "prokaryotes" but consider what we believe the most likely: the evolution of an already sophisticated LUCA, embedded in an intricate, promiscuous and multiphenotypic community, towards either of the three Domains. That it was a community played a capital role in the development of a unique genetic code at the earlier progenote stage [190] but was also a prerequisite for further evolution. The LUCA community probably consisted of several metabolic types [114], but a major heterogeneity in this population may already have been structural, i.e. the coexistence of phagocytic and non phagocytic cells [31], perhaps also of intermediary types (Jekely's "predators" [112] but without implying that this population contained anything like our modern "prokaryotes") This community was essentially dynamic and unstable, occupying a broad temperature range, and constantly incorporating or rejecting innovations via cellular exchanges, presumably by some merging process between cells devoid of rigid envelopes, unlike most prokaryotes (Fig. 2B).

Despite being a "community", this population would not in any way have escaped Natural Selection, the universal process that Dennett [191] called "Darwin's dangerous idea" and compared to a "universal acid" biting through everything, biological or not, perhaps the greatest philosophical advance since the dawn of mankind. We believe this should be emphasized because the concept of a "Darwinian threshold" [192] above which "vertically generated novelty can and does assume greater importance" (and species thus become recognizable entities) may be misleading, or at least ambiguous since the essence of Darwinism is often understood as Natural Selection, a process that must have operated at all stages of the emergence and evolution of life. Likewise – with all due respect for the evolutionary insights developed in "Collective evolution and the genetic code" [190] – calling "Evolution of the genetic code, translation, and cellular organization itself" a "Lamarckian process" also appears misleading by the implied suggestion of a basic difference between dynamic modes operating at different stages of the emergence of life. The inheritance of acquired characters (which is what Vetsigian et al. [190] are referring to as "Lamarckian" for emergence of a universal code in an evolving community) was accepted as a matter of course by Darwin himself in the absence of genetic knowledge. Yet, an essential point in Woese's theory is that "communal evolution" came, at all stages (and unavoidably), under Natural Selection.

This being said, the particularly heuristic character of the "communal" concept brought forward by Kandler [114] and Woese [9] to understand LUCA, its predecessor the progenote, and its descendants should be emphasized. The concept is a real breakthough in evolutionary biology thanks to its explanatory power. Just as, today, ecological interdependence makes it difficult to purify more than a tiny fraction of microorganisms out of any natural community, relentless competition took place between the highly interdependent organisms forming the LUCA community. Even the emergence of the ancestor of a particular Domain must have been an event conditioned by the actual state of ecological interactions (including viral invasions) occurring within the community at the time. Koonin [193] recently illustrated the power of the community concept in his "Biological Big Bang" model for major transitions in evolution, by discussing how genetic exchanges within an ancestral and promiscuous community could generate a large variety of forms from which new classes of entities (the new Domains) independently emerged at a new level of complexity. The substitution of dynamic communities for punctual origins in order to explain the emergence and divergence of biological and perhaps cultural trends (such as the origin of languages) may commend itself as a general principle.

From the above, it is clear that LUCA was a changeable entity; at the time of emergence of the first Domain it must have existed as a particular cell line that was the product of an untold number of genetic exchanges within the LUCA "community". As for other major steps in evolution [194], the emergence of the first Domain must have been the outcome of a crisis rather than a progressive development; today there is probably no situation anymore where the dynamics of evolutionary change is as powerful and as rapid. Whichever emerged first from the protoeukaryotic LUCA, the cell line that became the common ancestor of the two other Domains was certainly different and probably less promiscuous already. This type of evolutionary "eruption" would defy any attempt at reconstitution. We can, however, formulate a number of questions; few can be answered but an important point is that many of these questions could not have been formulated a few years ago.

### A simple model

The direct exploration of filiations by various phylogenetic approaches has delivered an enormous amount of information on the possible root of the tree of life; much of it is controversial but the concept of a protoeukaryotic LUCA developed here is not dependent of the branching order of the three Domains. Increasing doubts concerning an extreme- or hyperthermophilic LUCA, the pattern of apparently convergent evolution displayed by the lipid membranes of various thermophilic Bacteria and the comparison between membrane lipids in the three Domains support our hypothesis of a non-thermophilic (at the most moderately thermophilic or thermotolerant) LUCA community of protoeukaryotic cells from which Bacteria emerged by reductive evolution and Archaea by a "thermoreduction" implying the invention of *sn2,3 *isoprenoid ether lipids (Fig. 2B). The latter, with the ether bond as a selective, thus critical feature, are seen as the outcome of a molecular adaptation to extreme conditions that is in keeping with the alleged thermophilic origin of Archaea. Should however the archaeal Domain turn out to have emerged from a mesophilic or psychrophilic ancestor, other scenarios should be proposed. Even though the isoprenoid ether membrane is adaptable to the whole range of temperatures compatible with life, there is no obvious selective force to make such a structure emerge from a fatty acid lipid ancestor at low temperature, unless the ancestral archaeon had to adapt to another extreme condition, such as acidity, high alkalinity and/or an oxidating environment. Pursuing phylogenetic analyses of Archaea and their membranes is thus of great importance to test theories on their origin, and, by the same token, on the nature of LUCA.

Our working hypothesis for the emergence of the archaeal membrane is compatible with a bacterial [44] or an archaeal [45] root for the universal tree of life. The *sn*1,2-*sn*2,3 conversion in the emergence of archaeal membrane lipids could have been fulfilled in the precursor of Archaea before or after the virus-mediated RNA-DNA transition (fig 2). At any rate, what the chemical nature of biological membranes makes very unlikely indeed is that a full-fledged Archaeon would have been the common ancestor of both modern Archaea and Eukarya, since such a scenario would imply inventing twice the *sn1,2 *fatty acid lipid membrane, once for Bacteria, once for Eukarya. In this respect, we hope this discussion makes clear how important a major cellular feature may become when phylogeny remains contentious. Indeed, it was the analysis of membrane chemistry that suggested the idea that extreme thermophilic Bacteria emerged by convergent evolution from a non extreme thermophilic ancestor, itself derived from a LUCA community with a moderate but perhaps mixed temperature range, whereas Archaea at the outset adapted to extreme conditions by a shift in their membrane lipids.

Fig 2 summarizes our conception of the evolutionary steps in the rise and fall of LUCA, including the first increase in complexity discussed in the next section. Representing LUCA evolving within a communal population that would have "crystallized" [9] into different Domains thus aptly replaces the constrained "rooting" and "branching" metaphors of the past. In that sense, and in that sense only, do we see the necessity to replace the tree of the past by a new representation that however maintains Darwin's idea of a common ancestry for all living forms. Basically, nodes of divergence are replaced by an area stretching across time and space: one could compare the community embedding LUCA to a big tuber sprouting shoots and emptying itself in the process [49]. A visit to The New York Metropolitan Museum of Arts unexpectedly provided artistic support for this representation with Juan Miro's "Potato" (Insert A in Fig. 2).

## Emergence of life, complexity and order

We do not claim to treat in a comprehensive way the far reaching problematic that this title underlies. It seems however that the time is ripe to bring together a number of considerations that have been either left aside from many current discussions of the origin of life and LUCA, or neglected and sometimes misunderstood.

### The main steps between the emergent life and the advent of LUCA

Our focus will be the question of LUCA complexity as summarized in Fig. 2. We have seen above that LUCA may be understood as a diverse community of already metabolically and genetically sophisticated organisms. Its predecessor the progenote, more primitive and modular, was also a heterogeneous and diverse community of cells engaged in the emergence of a genetic code [190]. How did it come about? By using the word "complexity" we do not imply anything else here that an intuitive grasp of the concept. This is indeed enough to contrast two apparently unreconcilable views on the origin of the most important feature of living cells, the ability to replicate and to reproduce. The classical approach espouses the Lamarckian idea of evolution from the simple to the complex and focuses on experiments trying to reconstitute primordial RNA molecules capable of replication. The main merit of this approach is indeed to focus on actual experiments and some remarkable results have been obtained on the way some ribozymes may have started to replicate themselves [195,196]. However the emergence of self-replicating entities of increasing complexity requires both the formation of compartments (without which no distinction can be made between genotype and phenotype, and parasitic molecules can not be removed) and an ambient metabolism from which to draw renewable building blocks; such a metabolism therefore should be self-sustaining to a certain extent; de Duve [197,198] and Wachtershauser [104,199] have presented different versions of dynamic, evolving and self-sustaining metabolic networks.

Obviously, to make life emerge, some form of autocatalysis is required. The question is however, whether autocatalysis had to emerge as a primordial event already implying the replication of nucleic acid molecules (not necessarily the RNA we know today) or whether it was the global property of "catalytically closed" systems ([34,200] and other references below). The proponents of this alternative that radically departs from the classical one in its central argument have developed models that predict the emergence, before any molecular coding system, of entities that are both metabolically developed and autoreplicating (Fig. 2B). Kauffman ([34], see also Dyson [201]) discussed extensively how such a system could appear in a prebiotic environment where peptides [200] or both peptides and oligomers of nucleotides (both naturally endowed with catalytic properties) could be formed [34]. Above a certain level of diversification and catalytic interconnections, the system would undergo "catalytic closure", thereby becoming capable of self-replication (Fig. 2B). Using the related concept of "compositional heredity", Segre, Lancet and coworkers developed a similar model [202-204] and placed the emphasis on lipids, calling attention to the probable existence of several classes of "lipozymes" [203]. As these views are mutually compatible, it is tempting to merge them and to envisage the emergence of lipid-enclosed compartments enveloping a variety of ribozymes and peptides large enough to undergo catalytic closure (Fig. 2B). Indeed, among several types of entities *a priori *capable of catalytic closure, it is conceivable that only those combining lipids, RNA and peptides in an ambient protometabolism had a future because of the potential of nucleic acids to engender a coding system. To understand the emergence of such systems, perhaps one should also take into account what Ray called "sociality", referring to entities that can only replicate when they occur in aggregations [205].

Such lipid-enclosed compartments might have formed in very different environments: for example the relatively cool hydrothermal vents of the type found at the "Lost City," that combine the advantages of non-extreme temperatures with the capacity to concentrate macromolecules in pores [206,207] or, at the other extreme, glacial ice [208]. Wachtershauser has described how membrane lipids could arise from a surface metabolism developing on particles of pyrite in a volcanic setting [199]. For that matter, if we are to explain the emergence of self-replicating entities by catalytic closure of interactions between polypeptides, polynucleotides and the first amphiphilic, lipid-like molecules (Segre's "lipozymes" [203]), it appears reasonable to assume the latter were of protometabolic rather than meteoritic origin [209]. Admittedly, the "compositional" type of model suffers from lack of experimental support, which is not surprising considering the number of parameters, conditions and alternatives that would have to be explored in order to put it to experimental test. It has however the merit of considering the properties and interactions of real molecules, a prerequisite emphasized by Pross [210]. Catalytic closure has another, powerful scientific merit: it is *predictable*, something that cannot be said of most proposals for the origin of single replicators. However farfetched its application to biological systems may appear to some (see, for example, [210-212]), the hypothesis of a primeval role of metabolism originally devoid of a coding system in the emergence of the first precursors of living cells is gaining ground [213,214]. It has also the merit of presenting the emergence of a coding device culminating in the directed synthesis of the best possible catalysts – enzymes – as a process arising under the immensely efficient driving force of Natural Selection for greater fitness, by increasing the reproducibility of the autoreplicating system and eliminating unnecessary and parasitic reactions. In this frame of thought, still purely theoretical efforts "in search of the simplest cells" [215] predict how heredity could evolve in cross-catalytic, autoreplicating networks by virtue of minority components assembling into polymers [216,217].

Various experimental and conceptual advances [108,195,199,218-220] might all be considered in the frame of catalytically closed systems. The statement that "the evolution of nucleic acid replication and of nucleic acid-catalysed peptide synthesis must have been intrinsically linked" [108] is particularly interesting in this respect. In fact, catalytically closed, autoreplicating entities should be regarded as natural incubators, test-banks for the relentless selection and improvement of any type of molecular approximation of a coding system increasing the overall efficiency of the catalytically closed entities. In other words, the likeliness of a coding device emerging in the enclosed environment of such autoreplicating entities would be much higher than in a non- or poorly self-sustaining environment, the more so that these entities would be able to exchange material (again, the community paradigm). This view ceases to oppose "metabolism first" to "replicating first" by offering a synthesis between two models that indeed do not have to be irreconcilable. This goes well beyond the idea that the "emerging primal system was both metabolic and replicative" [210] by explicitly transfering the burden of emergence of the coding system to Natural Selection exerting its screening power on a self-sustaining, autoreplicating environment, thereby considerably increasing the chance for such a system to develop. We could therefore abandon the opposition between "metabolism first" and "replication first" and consider the emergence of genomes within the molecular environment of autonomous, replicating entities originally devoid of them as a sound alternative to current theories.

### Appealing aspects of the catalytic closure hypothesis

Catalytically closed systems such as proposed by Kauffman [34,200] have other merits still; they assume the prebiotic formation of peptides, a type of event considered as probable and anterior to the emergence of any self-replicating nucleic acid by de Duve [198]. This questions the hypothesis of a pure RNA world and makes de Duve' theory of a metabolism arising from protometabolism by selection and congruent with it, even more appealing than it originally was [197,198].

One of the most attractive aspects of catalytic closure as a possible step in the emergence of life is that it substitutes a qualitative jump toward a high degree of complexity for the laborious progression from simple to complex that used to be taken for granted but lost much of its simple appeal when molecular mechanisms came to be discussed. How complex such a catalytically closed entity possibly was has been discussed by Kauffman [34] who estimated that several thousands of catalytically interacting polymers (6,200 if the probability of catalysis for any reaction by a particular polymer is 10^-8^, 18,200 if this probability is 10^-9^) would have been capable of forming an autoreplicating system. Though no direct correlation can be suggested, it is nevertheless striking that the number of catalytic functions necessary to close the system (admittedly under somewhat arbitrary conditions) approximates the number of genes in a simple eukaryote. The primordial self-replicating entity was thus from the start a fairly complex one.

The above calculation was refuted by Lifson [211] who concluded that Kauffman's model was therefore invalid. This refutation was carried over in the literature [212] but we believe it may not be correct. Kauffman' mathematical treatment of such a complex problem is deliberately and utterly simplified, which already limits the validity of a too narrow refutation. More specifically, the treatment focuses on an alleged constant probability P that one polymer species has of catalyzing any specific reaction; according to Lifson [211], P = P'.P", P' being the probability that a polymer is a catalyst without specifying which reaction it catalyses, and P" the probability that a catalyst catalyses a particular reaction. P' is considered by Lifson to be a number "much smaller than 1" (which is however not obvious at all); this is then mathematically shown to ruin Kauffman's claim for "crystallization of connected metabolism as a percolation problem". The refutation would however be questioned if P' were not that low (how much was left out of Lifson's comments). In the same line, an earlier account of Kauffman's theory was considered by Joyce as "resting on a highly overoptimistic estimate of this probability" (see [211] Appendix A). At least the matter lends itself to experimentation and could be granted more consideration than subjective criticisms.

At any rate, catalytic closure is a powerful concept and other formulations of the same basic idea of replication without a genome have been presented, such as the models of Segre, Lancet and collaborators, [202-204] – integrating chemical kinetics – the model of Jain and Krishna [221], and more recent elaborations on compositional heredity [222,223] which take into account the environmental component, energy fluxes, various types of molecular interactions, and propose experimental tests. Experiments on mineral surface-directed membrane assembly and replication of bilayer-membrane vesicles pave the way towards reconstitution of cellular ancestors [224,225].

This catalytically closed entity was not just a bag of catalysts. A theory was recently proposed to explain how catalytic aggregations of increasing complexity (such as metabolic chains) may have formed spontaneously [35]. The importance of molecular complementarity in the formation of aggregates within such systems was also stressed by Hunding et al. [222]; this should have been an essential aspect of progressive metabolic organization. Moreover, "molecular crowding" is also recognized as an important factor in the formation of complexes and networks of proteins ([31] and ref therein). This type of "complexification" is an objectively defined process, contrasting with the vague idea of increasing complexity up the evolutionary scale that has been assumed by many as a matter of course since its formulation by Lamarck. The same can be said of considerations on functional information in complex emergent systems developed by Hazen et al. [36].

## Conclusion

The topic of LUCA's identity has evolved considerably in the last few years and we believe that a synthetic working hypothesis can be proposed that draws its strength from several advances (Fig. 2B). We summarize this synthesis in ten points. Our own contributions mainly concern points 2 and 5 to 9.

(1) The word "prokaryote" has become inadequate and misleading.

(2) Eukaryotic idiosyncrasies and a reappraisal of phylogeny do not support the notion of a prokaryotic LUCA nor of prokaryotic ancestors of the eukaryotic cell body. The order of branching in the universal tree has no bearing on the cellular architecture of LUCA.

(3) LUCA was a protoeukaryote, with a RNA genome inherited from its progenote ancestor. This RNA LUCA was in a metabolically and morphologically heterogeneous community, constantly shuffling around genetic material. Part of it was phagotrophic. LUCA remained an evolutionary entity, though loosely defined and constantly changing, as long as this promiscuity lasted.

(4) The RNA to DNA transition took place independently in different lineages of this community; the intervention of viruses appears a likely mechanism. This process led to the emergence of the three Domains.

(5) Within the LUCA community some cells developed membranes with the rudiments of electron tranport-driven phosphorylation; we suggest those cells (perhaps already engaged in reductive evolution) gave rise to Bacteria and Archaea.

(6) LUCA was a mixture of cells with different, overlapping thermal profiles (up to moderately thermophilic or thermotolerant); it had membranes with *sn1,2 *fatty acids lipids.

(7) Archaea arose by reductive evolution under selection for adaptation to high temperatures; this entailed the replacement of *sn1,2 *ester fatty acid lipid by *sn2,3 *ether isoprenoid lipids in the cell membrane, without major enzymatic reconversion.

(8) Bacteria arose by reductive evolution; secondary, convergent adaptations to thermophily entailed the formation of a variety of membrane lipids, often with ether bonds, but still with a *sn1,2 *stereoconfiguration.

(9) LUCA was genetically redundant; therefore the differential loss of paralogous gene copies in different lines of descent is a *predicted *source of phylogenetic discrepancies with respect to the SSU-rRNA tree. We expect a considerable proportion of these anomalies to have resulted from this process rather than from horizontal gene transfer, especially between Domains or distantly related taxa. Duplications occurring downstream from the initial divergence into Domains may also have contributed in no small measure.

10) The models of "catalytic closure" and "compositional heredity" transcend the old opposition between "replicators first" and "metabolism first" by offering a cradle for the relentless selection of a genetic code in a favourable environment. A previous refutation is shown to be inconclusive. These models have the advantage of being predictive and therefore testable experimentally.

## Competing interests

The authors declare that they have no competing interests.

## Authors' contributions

BL devoted himself to the phylogeny and genetic constitution of LUCA, YX to the question of membrane adaptation in thermophily. NG conceived of the study after a thorough bibliographic search and wrote the first draft of the paper. All authors read and approved the final manuscript.

## Reviewers' comments

### Reviewers' report 1

Anthony Poole, Department of Molecular Biology & Functional Genomics, Stockholm University, Sweden – currently at: School of Biological Sciences, University of Canterbury, New Zealand

The main point of this paper is to put forth a scenario for the nature of the Last Universal Common Ancestor (LUCA) as a complex, protoeukaryotic lineage with an RNA genome and nuclear compartmentation. If I understand correctly, the argument is built on the proposal, adapted from Woese's Universal Ancestor model (ref. 9 in the paper) that there was an extremely diverse community of cells from which the three domains emerged. Woese's model is that rates of horizontal gene transfer were extremely high early in the evolution of life, and that transfer rates became lower with time, eventually leading to 'crystallisation' of the three domains independently from this early state. As far as I can tell, the authors are comfortable with this interpretation, and take this scenario as their starting point. However Glansdorff, Xu & Labedan's model diverges considerably from other published scenarios in several important ways. First, while they appear happy with high rates of gene transfer very early, they see a minor – perhaps marginal – role for horizontal gene transfer after 'crystallisation' (the term Woese uses in his model to refer to the distinct emergence of lineages which have become refractory to further gene transfer). Glansdorff and colleagues also argue that reductive evolution and themoreduction had an important role in shaping bacteria and archaea, respectively, and that, because the eukaryote lineage is argued not to have been subject to such reductive evolution, many traits within this domain are ancestral (read: features of the LUCA). A central part of their model is that differential gene loss has played a major role in shaping the differences between the three domains of life.

The authors succinctly summarise their opinions in ten points in the final conclusions section, which is much appreciated, since it is an unwieldy manuscript at times (though there are some wonderful insights among some more well-trodden material – the quizzical air in which zygogenesis is brought up (why haven't proponents of fusion been all over this?) and the anticipatory remarks that serve to castrate any use of this observation (don't even think of trying to stretch it that far), make for informative reading). All of these are interesting points; some have been made before, and some are updated and extended here. There are certainly some contentious ideas among these which many workers will find difficult to accept.

I separate my review into more mundane editorial material and material I think warrants discussion.

I think the manuscript could be shortened, and is in need of editing for language. I recommend shortening the discussion of the term 'prokaryote', and think it is better to incoporate one or two brief points into one of the other sections, rather than devote an entire section to what is fast becoming a rather tiresome semantic debate. The section 'Eukaryote gene promoters...' is, on the other hand, so short as to be uninformative. Please either review this material so that the general argument can be understood, or delete it.

*[Author's response: We have trimmed the paper in places, but, on the other hand, we had to expand on some questions in order to answer the various comments of the referees. As regards the fallacies and pitfalls of the term "prokaryote", we think that it is appropriate to maintain the core of the discussion as a separate item; we feel indeed that the matter is more than semantic, as appears from P. Forterre' s comments also (see further). The section on Eukaryotic promoters has been made more explicit]*.

Concerning the section on 'Paleochemistry', I have heard that concern has been expressed by one of the authors of the Brocks et al. study (ref 61) that the lanostane was a contaminant. Tellingly, Brocks does not cite that original paper in a recent review on using molecular biomarkers to examine deep evolutionary history (Brocks & Pearson 2005, Reviews in Mineralogy & Geochemistry, 59:233–258), but I have also heard that the contaminant verdict is not universally shared by all authors. This verges on gossip, but it might be worth contacting the senior authors of that study (Brocks, Summons) to ask what their current opinions are.

*[Author's response: You are quite right as JJ Brocks himself confirmed to us and discussed in detail in a later study (new reference). We therefore rephrased our original discussion by drawing attention to the current controversy and mentioning Brocks'alternative interpretation of the presence of sterol biosynthetic pathways in several bacterial groups (horizontal transfer from a protoeukaryote or early eukaryote into ancient bacterial lineages)]*.

The section, 'Emergence of life, complexity and order' seems a bit vague, and quite disconnected from the rest of the manuscript. If it were my manuscript, I would cut this section out.

*[Author's response: We would however like to maintain this section because we feel the necessity to bring together fields that up to now seem to have developed almost in mutual ignorance. We feel the concepts of catalytic closure and compositional heredity place the emergence of life in a logical perspective; moreover we think the community paradigm is not only useful to explain the emergence of the code but also essential to understand how a pregenomic phase may have developed. Finally, Lifson's refutation of S. Kauffman's model (ref 211) that has been carried over in the literature appears to us inconclusive, something that had to be stressed as well]*.

I now turn to the key ideas which I think warrant a response.

For Glansdorff and colleagues, the puzzling gene distributions that many have attributed to horizontal gene transfer are better explained by differential loss of paralogues. This, they argue, explains most incongruence between phylogenetic trees. So, rather than rejecting the tree of life outright on the basis of concluding that horizontal gene transfer is far too prevalent (as some have recently advocated) these authors instead argue that their model (gene loss) predicts the patchy distribution of genes and traits that is observed.

This is a rather bold stance to take, and is interesting insofar as it represents the other end of the spectrum from the proposal that there is no tree of life. Personally, I don't subscribe to either extreme scenario, though there is certainly room for both *mechanisms *(i.e. gene loss and horizontal gene transfer). Glansdorff et al, while clearly disliking the arguments in favour of extensive, ongoing transfer, nevertheless concede that, in most cases, it would be difficult to distinguish between loss or transfer. Indeed, given that we have no good scheme for weighting the relative probabilities of multiple losses of cryptically paralogous functionally redundant genes versus distant gene transfers, this is true. For both mechanisms, there are good examples, but the argument here is whether most cases can be explained as being down to one mechanism or the other. Confidence probably does run a bit on the high side among some proponents of extensive horizontal gene transfer, at least judging by some of the more extreme statements that have been made in the recent literature, but is the other extreme any more informative?

*[Author's response: We certainly do not want to give the impression that we deny the occurrence of any horizontal gene transfer (HGT) but the very wording of the title announcing the section on LUCA genetic redundancy may have suggested that we did; it has been amended to avoid any misunderstanding. The essence of our argumentation is that multiple losses of paralogous gene copies present in the ancestral gene pool of the LUCA community may be responsible for much of the incongruencies observed in genetic trees because it is a prediction. Moreover, the occurrence of duplications during intra domain evolution is also expected to contribute. However, in much of the literature dealing with phylogenetic incongruencies, HGT has been the only explanation considered and those who advocated caution and restraint in this respect have found but little echo. We therefore felt the necessity for some refocusing. Our view is however not "extreme" in that it does not reject the occurrence of HGT. Since however both you and P. Forterre felt that our original formulation perhaps unduly minimized the importance of HGT, we modified the text by mentioning a few instances of interdomain HGT pointed at by Forterre in his comments; these are instances where natural selection can be invoked as a driving force (which is not the case for the plethora of alleged HGTs), such as the contribution of reverse gyrase to the emergence of hyperthermophily, something we already had mphasized in a previous publication (ref 29). This however does not detract from the view that such instances appear to be rare and that many incongruencies could be more parsimoniously explained by differential loss of paralogues. We therefore do not see ourselves as proponents of an extreme view; as you point out, both mechanisms must have contributed to the present situation but a conceptual readjustment appeared necessary]*.

It is in a sense an irony that the model Glansdorff et al. present, which reads as being fairly hostile towards gene transfer (at least between domains), is nevertheless entirely dependent on it. By favouring Woese's scenario of high rates of gene transfer prior to the emergence of the three domains, Glansdorff et al. can have their cake (or in their case, potato) and eat it too – a totipotent ancestral community of genes but without the burgeoning genome size. I will limit myself here to the following point: while Woese's ideas have become popular, there really is no evidence for higher rates of gene transfer early (i.e. pre-three domains). I for one have a hard time seeing how this scenario of one interconnected gene pool with unconstrained gene transfer fits with our understanding of biological systems. I am fine with gene transfer between modern lineages, but less convinced that the hypothetical scenario Woese proposes has any firm basis in biology and likewise concerned that it is sufficiently vague as to permit rather divergent interpretations. If we need to accept one speculative scenario to accept the other, the second can come down like a house of cards. Better then to argue for a role for the mechanism of gene loss, though not in this extreme manner. Seen in this light, their description of the carbamoyltransferase dataset is useful, but I think it might help if the authors were to address the relevance of this single example of paralogous gene loss more explicitly and in more detail; i.e. how much more of the data do they think can be explained unambiguously as losses? The authors do cite a handful of examples, but those who argue for a significant role for horizontal transfer make bold statements, as embodied in the phrase, 'the tree of one percent' coined by Dagan & Martin (2006; Genome Biology 7:e118).

*[Author's response: We see no contradiction in relying on genetic promiscuity in the pre-domain era while considering that lateral gene flow must have been considerably reduced after the "crystallization" that generated the three Domains from LUCA. Of course, later on, the development of mobile elements of various kinds must have allowed exchanges to occur regularly, mostly between related members of the same domains. But we stress that it is misleading (see text) to infer the general amplitude of horizontal flow from the identification of putative foreign (but mostly bacterial-like) genes in E. coli. As already explained above, we do not want to appear as arguing for gene loss in an extreme, exclusive manner but we emphasize that it is a prediction of the genetic redundancy of the LUCA community. For the reasons exposed by Woese and coworkers in ref 190, we are convinced by the necessity of a communal phase preceding Domain divergence in order to explain the emergence of a universal genetic code. It appears much more appealing to conceive of such a development in a promiscuous community, where eliminations of false starts, corrections, improvements and new combinations can proceed under constant selection, rather than in an isolated cell line. This could also be valid for the developments of other biological systems. As regards the carbamoyltransferase data set used to illustrate the model of differential gene loss, some additional comments have been included. In fact, our early analysis (ref 29, 172, 173) pointed the way to the systematic identification of paralogous gene copies in the LUCA community; this needs to be followed up rather than ignored]*.

A novel proposal that Glansdorff et al. present is the idea that LUCA (or some subpopulation of the 'community') may have been nucleate. The implication here is that LUCA possessed a nucleus, and that this was lost from the archaea and bacteria, with a handful of lineages perhaps retaining this cellular architecture (notably the Planctomycetes). I certainly agree that some current explanations for the origin of the nucleus are insufficient and have myself published critiques of some of these recently. I also agree that, formally, we cannot actually tell when the nucleus arose, since we cannot readily distinguish between multiple losses from an earlier ancestral nucleate state and a single gain in the lineage leading to modern eukaryotes. So, yes, it is entirely possible that LUCA had a nucleus, but currently there is not a shred of evidence that can be brought to bear on that possibility. I think that Planctomycetes and other 'prokaryotic' lineages are fascinating, but until we can establish whether their internal membrane structures are homologous or analogous to those in eukaryotes, one cannot really use them as leverage to favour an ancestral nucleate LUCA. I get the impression that this is an ulterior motive behind the paleochemistry section, i.e. citing the Pearson et al. paper (ref. 63) on sterol synthesis in Planctomycetes. Again, I am not so sure the data as they are can be used to argue for vestigial traces of a protoeukaryote LUCA among the biology of modern planctomycetes. If there's one thing I would like to know, it is the authors' opinion of the model published by Devos et al. (2004; PLoS Biol 2:e380), which is, to my mind one of the most interesting models for the origin of the nucleus, and should be addressed in any discussion of the origins of this organelle.

*[Author's response: The possibility of a protonucleus in a RNA LUCA is of course nothing more than a conjecture (as many developments in this field) but, as mentioned in the text, it is in keeping with Forterre's proposal on the emergence of DNA genomes via the intervention of viruses and it seemed appropriate to present our view in the perspective of this heuristic theory. As regards the interpretation of the Planctomycetes "nucleus" your comments converge with those of P. Forterre since we can not tell presently whether it is analogous or homologous to those found in eukaryotes. There was in our previous version of this manuscript some comments on this topic, which we have now reintroduced to take this question into account. We are happy to include a discussion of Devos et al, which we indeed feel is particularly relevant and even illuminating. In fact, Devos'model for the endogenous emergence of the nucleus and the endocytotic apparatus by recruitments of particular protein domains suggests how nuclei may have appeared in the protoeukaryote, perhaps even repeatedly (for example in Planctomycetes). It is of course also relevant to the origin of phagocytosis; in fact, it makes this remarkable capacity appear definitely less mysterious and, in our view, supports the notion that it emerged early, perhaps already among the LUCA community, as argued in the first version of this paper]*.

I do agree that some features of modern eukaryotes are likely to resemble equivalent features in LUCA, and that some features of modern eukaryotes more closely resemble the ancestral state than do prokaryotic equivalents. This is of course just normal pedestrian evolutionary biology, and, as Glansdorff et al. clearly state, Darwinian thinking (i.e. not presuming, a priori, to know the ancestral state, and, accepting, that we cannot, a priori, ascertain in which lineages we might find the ancestral state, if at all) is preferable to applying Lamarckian notions of 'progress'. However, I do not think this means that we can necessarily extrapolate to other features of eukaryotes, such that 'protoeukaryotes' become the ancestral state. In this regard, I disagree with Glansdorff et al. in that I do not see any evidence that LUCA (or some subpopulation) was phagotrophic. Certainly, this is possible (and once again I am in principle open to this possibility), but, as with the nucleus, there is to my knowledge no evidence that can be used to support such a contention.

*[Author's response: See comments above]*.

The other ideas described in this paper, as summarised in the conclusions as a set of 10 points, either draw from the work of others, or from the authors' own published work, and I think there is no need for me to comment particularly on this synthesis – I feel disinclined to critique the critique, or advocate the advocacy (or the possible variants thereof), as I think it would largely just serve as a reflection of my own opinions. Overall, I think this synthesis makes for interesting reading, though doubtless there is something in here for everyone to disagree on – such is the nature of the field!

### Reviewers' report 2

Patrick Forterre, Institut Pasteur, Paris, and Institut de Génétique et Microbiologie, Université Paris-Sud, Orsay, France

In this paper, Glansdorff, Xu and Labedan synthesize, update and summarize the state of the art concerning the nature of LUCA, from the viewpoint favouring a rather complex proto-eukaryotic LUCA. This viewpoint, although at odd with most current thinking, is supported by many arguments often overlooked in most evolutionary papers on early cellular evolution. The merit of this paper is to explore this possibility in a very exhaustive way. Being myself an early proponent of a proto-eukaryotic LUCA, I have of course a favourable prejudice for most hypotheses presented here, and I strongly support publication of this paper.

A very important point made by the authors is that the rooting of the universal tree in the bacterial branch does not prevent a proto-eukaryotic like LUCA, since the "prokaryotic" phenotype of Bacteria and Archaea might have originated twice independently by streamlining from the two different nodes of the universal tree. By the same reasoning, LUCA might also have been prokaryotic-like, even if the root turned out to be in the eukaryotic branch. These are quite obvious remarks for someone used to work with a correct evolutionary background, but this is not so clear for most biochemists and molecular biologists. I should confess that I have been myself misled by the wrong idea that the bacterial rooting implied *de facto *a prokaryotic-like LUCA. This, together with my old prejudice for a proto-eukaryotic like LUCA, explains why I scrutinize so deeply the data supporting the bacterial rooting. This is not to say that solution of the rooting problem is not important, but in any case, it will not solve the problem of the nature of LUCA (except if the root turned out to be within one of the three domains, something highly unlikely). The great challenge is to polarize the characters that are common to Archaea and Eukaryotes, are they primitive or shared derived traits? In the absence of an outgroup, this is a very difficult task.

There are other mistakes currently made in the interpretation of phylogenetic trees and the authors make one of them (a very classical one) when they conclude that the hypothesis of an hyperthermophilic ancestor for bacteria has been weakened when the positions of hyperthermophilic bacteria (*Aquifex, Thermotoga*) as the two earliest branching lineages in the bacterial tree have been put into question. This is based on the wrong assumption that the phenotype of a modern organism was already present at the base of the lineage leading to this particular organism. This assumption is safe only if several basal lineages share this phenotype. For instance, the hypothesis that the last common ancestor of Archaea was a hyperthermophile was for a long time supported by the fact that all basal archaeal lineages were ONLY populated by hyperthermophiles. In contrast, *Aquifex *and *Thermotoga *belong to phyla that also include mesophiles and moderate thermophiles, such as *Geotoga *or *Hydrogenobacter*. As a consequence, even if these phylaare the two earliest bacterial branches (which is probably not the case) this would not automatically lead to the conclusion that the last common bacterial ancestor was a hyperthermophile. Similarly, it seems now that the first basal branch of the archaeal tree lead to mesophilic (even psychrophilic) archaea (ref in the manuscript), but this does not imply that the last common ancestors was mesophiles or psychrophiles

*[Author's response: You are of course perfectly right and we hope to have cured the text of any misleading statements in this respect]*.

I agree with Carl Woese, Norman Pace and the authors that the term prokaryote is misleading. The authors refute the proposal of Koonin and colleagues to base the term prokaryote on the translation/traduction (TT) coupling, because some uncoupled TT should occur in bacterium of the phylum Planctomyces, such as *Gemmata obsuriglobus*, in which DNA is separated by a nuclear-like membrane from most ribosomes. This is a weak argument, because TT coupling could occur in the nuclear compartment of *G. obsuriglobus *which also contains ribosomes (Fuerst, 2005). For me, the TT coupling cannot be used as a positive trait to group Archaea and Bacteria, because we don't know if the TT coupling is a convergent or a homologous trait, and, in the latter case, if the TT coupling is an ancestral or a shared derived character. Only in the last case, the TT coupling could be considered as a synapomorphy, eventually justifying a common name for Archaea and Bacteria.

*[Author's response: We agree that the mere mention of a nucleus-like structure in Gemmata is only a weak argument and we refer to your precisions in the modified text]*.

Finally, as noted by the authors, the term prokaryote (before the nucleus) is not neutral since, it gives the false impression that the ancestral nature of prokaryote is well established. I suggested once to use the neutral term akaryote (without nucleus) if one really want a name to distinguish Archaea and Bacteria from Eukaryotes on a structural basis (Forterre, 1992 [Forterre, P. Neutral terms. *Nature*, 1992, **335**, 305]). In that case, most bacteria would be indeed akaryotes, except those, like *Gemmata obsuriglobus *and Poribacteria, which are synkaryotes (with a nucleus) and not eukaryotes (to say that a *G. obscuriglobus *is a eukaryotic bacterium would be probably confusing).

*[Author's response: Again, we agree; a new nomenclature is badly needed to avoid current misconceptions]*.

The problem of the origin of the nucleus of Planctomycetes is probably too much emphasized by the authors (and sometimes ambiguously) as possibly homologous to the eukaryotic nucleus (i.e. they share a common ancestry) and testifying for the situation in LUCA. There is presently no indication that the nucleus of some Planctomycetes is homologous to our nucleus. It should be mentioned that, if all Planctomycetes have a system of internal cytoplasmic membranes, the intracytoplasmic membrane (ICM), only *Gemmata *species have a true nucleus (i.e. an invagination of the ICM that separates the nucleoid from a portion of the cytoplasm that contains ribosomes) (Fuerst, 2005 [Fuerst, JA. Intracellular compartmentation in planctomycetes. *Annu Rev Microbiol*. 2005, **59**, 299–328]). The nucleus of *Gemmata obscuriglobus *thus could be a recent invention in Planctomycetales. It will be especially important indeed to solve this question in studying extensively the nucleus of Planctomycetes and Poribacteria. For me, the discovery of these bacterial nuclei indicates that the formation of such structure might have occurred several times independently in the course of evolution. In any case, it is quite fascinating to consider the similarities between bacterial and eukaryotic nuclei, in both cases the nuclear membrane is produced by the recruitment of an internal cytoplasmic membrane system, the ICM and the endoplasmic reticulum, respectively.

*[Author's response: Our presentation of the Planctomycetes was indeed biased in the sense that we did not explicitly mention the notion that the nucleus of Gemmata is not necessarily homologous to the eukaryotic one. In a previous version of the draft there was a section dealing with this problematic; in keeping with your comments and those of Dr Poole, we have reintroduced these considerations in the present version]*.

The authors suggest that RNA-cells themselves might have had a nucleus. I think this is a real possibility and, by the way, RNA nuclei still exist today. In a recent review, Miller and Krijnse-Locker (2008) [Miller, S. and Krijnse-Locker, J. Modification of intracellular membrane structures for virus replication. *Nat Rev Microbiol*. 2008, **6**, 363–374] remind us how modern RNA viruses recruit membranes and vesicles from the endoplasmic reticulum to form viral factories in which their replication apparatus is surrounded by one or two membrane layers, with an opercule for communication with the cytoplasm (pore?) (a true RNA nucleus!). I think therefore likely that both akaryotic and synkaryotic RNA-cells were present in the RNA world.

*[Author's response: Thank you for this information; we have now included this reference]*.

On page 5, the authors state that *"the fact that the eukaryotic cell is more complex than modern prokaryotic cell is largely the result of the endosymbiosis *(of mitochondria) *not of a basic trend*". I don't think this is correct. I would say that it is because the proto-eukaryotic cells were already more complex than modern akaryotes (in particular capable of phagocytosis) that they have been able to enslave a bacterium to become progressively a modern eukaryotes. The more complex and mysterious part of the eukaryotic gene expression machinery is the spliceosome and I don't think that it emerged as the result of endosymbiosis. Indeed, as the authors notice themselves, it should have take a long time for the emergence of a complex spliceosome, refuting the idea of the sudden origin of spliceosomes from group II introns shortly after the mitochondrial endosymbiosis. This is for me a very convincing argument. Also why group I introns present in archaeal and bacterial genomes did not invade the eukaryotic nucleus?

*[Author's response: This is a useful remark indeed; we have taken it into account in the present version]*.

For me the spliceosome (not the nuclear membrane) is the real hallmark of the domain Eukarya. All Eukarya have spliceosomes and it is absent from all Archaea and all Bacteria. Furthermore, it really points to the heart of the gene expression mechanisms. I would suggest renaming the domain Eukarya to Splicea and corresponding cells to spliceotes, instead of eukaryotes. Indeed, it's a very anthropocentric view to consider that our nucleus is the true (eu) nucleus. Does this means that *G. obscuriglobus *is a false or incomplete nucleus? I would thus say that Spliceotes and the bacterium *G. obscuriglobus *are two syncaryotes that belong to different domains with nuclei. At our present state of knowledge, these nuclei might be either homologous or analogous. I would like to read the comment of the authors on this nomenclature question. They might have ideas of better names?

*[Author's response: In our manuscript we only focused on the problems raised by the term "prokaryote" but of course, if we want to drop it, it is the very duality "prokaryote-eukaryote" that is brought into question and the nomenclature you propose would be an adequate and radical departure from the present situation]*.

Coming back to LUCA, the authors mention the paper of Delaye et al., on the monophyletic origin of ribonucleotide reductase (RNR) as an argument for a LUCA with a DNA genome. Delaye et al have missed the point here. There are three classes of RNR and they are distributed in the three domains in such way that none of them is universal. It is therefore possible that RNR were introduced later on (from viruses??) in the different lineages. Of course, one, two or the three RNR might have been present in LUCA (or in its community see below) and differentially lost thereafter, but this is only hypothetical and cannot guaranty us that LUCA had a DNA genome. By the way, the question of the real monophyly of the RNR activity is still an open question. The three classes of RNR are built around the same homologous core and use basically the same reaction mechanism, but they share this characteristic with pyruvate formate lyase (Stubbe, 2000 [Stubbe J. Ribonucleotide reductases: the link between an RNA and a DNA world? *Curr Opin Struct Biol*. 2000, **10**, 731–736]), and the core itself cannot have RNR activity. In each class, the RNR activity is obtained by association of this core with different protein folds, domains or subunits, and the use of different cofactors. As a consequence, one cannot exclude the possibility that the RNR activity has been invented three times independently, using the same basic protein core, misleading us to believe that RNR activities are homologous.

*[Author's response: We have referred to your comments when discussing the paper by Delaye et al]*.

I like very much how the authors discuss the problem of the temperature at which LUCA was living. In particular, the idea that one should take into account the notion of temperature range is welcome, as well as their critic of the Gaucher et al paper. All present data indeed support the notion of a mesophilic or moderately thermophilic LUCA and a secondary adaptation to Archaea and some Bacteria to hyperthermophily. The authors emphasize adaptation to hyperthermophily as the major selection pressure for the formation of the archaeal domain by thermoreduction. The question is more open now, with the discovery of the basal position of mesophilic Thaumarchaea and one cannot exclude that archaeal lipids originated in a mesophilic environment, but turned out to be well suited to allow some Archaea to explore rapidly the hottest environments. The replacement of ester bonds by ether bonds and the formation of monolayer lipids is indeed favoured by high temperature, but I don't see what selection pressure favoured *sn2,3 *versus *sn1,2 *lipids at high temperature. I would like very much to read the comment of the authors on this point. By the way, we possibly focus too much on the relationships between the two kinds of glycerolipids now present in the biosphere, it might be that the variety of lipids was much higher before and at the time of LUCA, and that the two types of lipids that we know have been selected randomly because they were those present in the three successful lineages at the origin of the modern domains. Anyway, I agree with the authors that it would be important to do experimental works on this issue by engineering new organisms with mixed lipids for instance.

*[Author's response: Indeed, the primary divide between sn1,2 and sn2,3 lipids may not be the direct result of selection. However, what we suggest was selected for in the course of adaptation to high temperatures was the recruitment of isoprenoid lipids and their ether linkage to a glycerol phosphate molecule. This almost certainly did not happen simultaneously and in the revised version we propose a more detailed scenario where isoprenoids (an ubiquitous type of molecule) were brought into the formation of membranes in a first step, to be followed by the formation of an ether bridge and then by the introduction of the sn2,3 conformation as a consequence of known properties of GGGPS. Perhaps there was (or still exists somewhere?) a membrane with ester isoprenoid lipids linked to G3P and/or ether isoprenoid lipids linked to G3P. The point we wanted to stress was that selection has probably been at work, a notion that is supported by the presence of ether bonds in both Archaea and thermophilic Bacteria. A salient point about our scenario is that interesting evolutionary experiments could be carried out with GGGPS]*.

The authors are dubious about the hypothesis proposed by several evolutionists of mixed membranes in LUCA, with both *sn2,3 *and *sn1,2 *lipids made by a non enantio-specific enzyme. The concept of a "non enantio-specific enzyme" is strange for me, and to my knowledge, has never been really discussed. In my opinion, as soon as you have a catalyst using a three-dimensional active site, you have no choice other than to select only one enantiomer for substrate and the product will be also enantio-selective. As a consequence, I don't think that non enantio-specific protein enzyme or ribozyme ever existed! The emergence of 3D catalysts explains indeed very simply why all protein amino-acids and nucleic acid sugars are of one enantiomeric form. Again, I would really like to read the comment of the authors on this question.

*[Author's response: We could not agree more; stereospecificity is a salient feature of enzymes, even if some exceptions are known; this comment has now been elaborated upon in the text]*.

I completely agree with the authors that the importance of LGT has been over emphasized, and that the notion of a web of life instead of a tree of life is misleading. However, although there was certainly a relatively high level of redundancy in LUCA and its companions (as in modern organisms), I have the feeling that the authors tend to go too far in refuting the existence of LGT between domains. They are many clear-cut evidences for such transfers. They are indeed rare and usually easy to detect through phylogenetic analyses, so that they cannot confuse species tree when analyses are properly done. The rarity of LGT between domains is well illustrated by the quasi-absence of exchange between archaeal Topo VI and bacterial Topo IV, two enzymes that have exactly the same catalytic activities. Only three out of more than 500 sequenced bacterial genomes have acquired an archaeal Topo VI, and none of the presently archaeal genomes has a bacterial Topo IV. Although rare, LGT exist and can be easily identified (the three Topo VI found in Archaea branch within Archaea in Topo VI trees, whereas the DNA gyrase found in Archaea branch within the bacterial sequences in DNA gyrase trees). Furthermore, they can have a profound influence in the history of a particular lineage. For instance, the transfer of reverse gyrase from Archaea to some bacteria has probably helped their adaptation to extremely high temperature whereas the transfer of gyrase from Bacteria to some archaea has profoundly changed the internal topology of their DNA. Therefore, in my opinion, one should have a more balanced view than the authors on the importance of LGT *versus *paralogy/loss in global evolution and more work is required to evaluate the relative importance of these two phenomena. I think for instance that paralogy/loss is overemphasized in the work of Ouzounis and colleagues leading to a LUCA with 1000 gene families.

*[Author's response: As already explained in our answer to Dr Poole, we do not wish to trace all phylogenetic incongruencies to loss of paralogues and we do not deny the occurrence of LGT (or HGT) but, as one of us already mentioned in a previous paper (ref 29), we stress that assuming an interdomain LGT (rare as you also emphasize) does not gain much credibility from the mere occurrence of a phylogenetic anomaly but well from the possibility of selection, such as in the case of reverse gyrase. Our view is in fact epistemologically "balanced" in the sense that it emphasizes an hypothesis (loss of paralogues) that has been systematically neglected even though it is a prediction. In other words it needs to be "falsified" rather than neglected]*.

Paradoxically, although the authors are strong critics of the overwhelming-LGT theory, they have adopted the viewpoint of a communal LUCA which, historically, has been proposed partly to take into account this theory (see Woese [Woese CR. Interpreting the universal phylogenetic tree. *Proc Natl Acad Sci U S A*. 2000: **97**, 8392–8396]). I am not convinced, as the authors are, by the heuristic character of the concept of LUCA as a community of organisms. If cell divided by fission from the time of LUCA, one is obliged to accept the existence of a single organism as the LUCA, as we are obliged to accept from population genetic the idea of a single women (the African Eve) at the origin of all women present on Earth today. Of course, LUCA was not living alone at that time (as the African eve herself) but among many other organisms that have no descendants today. Therefore LUCA was living among a community (that we can possibly call the LUCA community) but this community (Eve's village) should not be confused with LUCA itself. Unlike the authors, I thus definitely think that LUCA has been an identifiable cell line. For me, the community concept is only helpful to fight the very naïve idea that LUCA was the only cell at that time!!! Since LUCA was not the first cell, it could not have been alone. The community concept can be misleading because – associated to the "overwhelming LGT" theory – it can lead to the conclusion that all members of this community were quite similar to each other, exchanging genes as crazy (mimicking an ideal Hippy community). On the contrary, the authors make a good point in describing a quite diverse LUCA community, with various organisms using different metabolism and more generally different ways of life (possibly with both akaryotic and synkaryotic RNA or primitive DNA cells of various sizes). One thing I would like to add in the debate is that I see no reason why the contemporaries of LUCA should have been confined to a limited location on our planet (a single chimney in the more extreme scenario). All complex RNA and/or primitive DNA cells (with their viruses) living at the time of LUCA might have already colonized all the habitable biotopes of our planet, much like modern microbes, forming many diverse communities, only one of them including LUCA. They were probably limited or no exchange at some time between isolated communities, leading to speciation and later conflict in future encounter. Again, one can draw a parallel with the village of Eve, which was probably surrounded by other villages in a particular region of Africa and many more in all Eurasia.

*[Author's response: Your comments go straight at the heart of the matter! We are however wary of too close a comparison between LUCA and the "African Eve" because of the biological constraints of human sexuality and also because of possible misconceptions. Several geneticists (see for ex de Duve in "Singularities"ref 94) have pointed out that "Eve" was not the only woman to bear children who are ancestors of living people (their nuclear genes are still with us); the concept only illustrates a bottleneck of direct female ancestry passing through one individual. Besides, the time of the common ancestor of all males is quite different. Also, within the community of human geneticists, there is no agreement regarding the possible occurrence of interbreeding between the ancestors of H. sapiens sapiens with other groups of humans, even after the bottleneck. Coming back to LUCA, we agree that it must have existed as a particular cell line at the time of the major transition that gave rise to the first Domain but if the latter emerged in a very promiscuous community, it must have been the product of an untold number of genetic exchanges and it is conceivable that it was the result of a relatively short evolutionary « crisis »; today there is probably no situation anymore where the dynamics of evolutionary change is as powerful and as rapid as it may have been at the time of LUCA. We have included some comments to that effect in the revised version*.

*As already stressed in our answer to Dr Poole we feel very much in favour of Woese's community concept to explain the emergence of the code and of other basic biological systems; only thereafter could well defined, genealogically identifiable lineages have emerged as the result of both selection and drift, becoming more and more restricted in their possibilities of genetic exchange (Woese's "crystallization"). It is of course possible that during this period of increasing individualization, some important features were still exchanged, but we are definitely questioning the persistence of interdomain promiscuity after this stage. There is, by the way, some inconsistency in assuming both the occurrence of Domain "crystallization" and "widespread, indiscriminate" post-crystallization HGT of aminoacyl tRNA synthase genes (Woese et al.2000, **64**:202 and ref 2). We are happy to note that you have no objection to the concept of a phenotypically diversified LUCA community containing phagocytic organisms (see our answer to Dr Poole regarding Devos' model for the emergence of the nucleus and the endocytotic apparatus)]*.

I agree with the authors that Darwinian evolution operated early on, well before LUCA and the origin of the three Domains. The notion of a Darwinian threshold can be interpreted as opening the way for a Lamarkian view of early evolution (usually favoured by astrophysicists and chemists who are often dominant in number in the origin of life community). The authors should be aware that the notion of a "communal LUCA" can have the same effect, if one envisions the evolution of this community driven by an internal tendency for matter to evolve towards complex structures under physical principles that remains to be discovered. Physical principles can create the framework for biological evolution, but the motor will remain descendent modifications with natural selection.

*[Author's response: The "communal LUCA" concept has indeed raised some caveats (see for example de Duve in "Singularities"ref 94) and this is the reason why we have stressed the importance of natural selection as an unavoidable process. This would remain true even if there were "an internal tendency for matter to evolve towards complex structures under physical principles that remain to be discovered"; S. Kauffman (34) has shown how, in theory, the spontaneous formation of ordered structures would be submitted to natural selection in a way that is bound to create novelty and further structuration when selection operates at the border of chaos; in this very heuristic and penetrating theory, the role of natural selection remains absolutely determinant]*.

Finally, since the authors often refer to the "three RNA cells-three DNA viruses (3R/3V) hypothesis", I will say that more recently, I tend to come back to a simpler "two RNA cells-two DNA viruses hypothesis (2R/2V)". For me, the interest of the 3R/3V hypothesis was to explain the existence of three well defined versions of ribosomal and other universal proteins, and also to explain some critical differences between the DNA replication apparatus of Archaea and Splicea (Eukarya), in particular their very different sets of DNA topoisomerases). The last point has been weakened by several recent findings suggesting that Topo VI and Topo IB could have been possibly already present in the common ancestor of Archaea and Splicea (Malik et al., 2007 [Malik SB, Ramesh MA, Hulstrand AM, Logsdon JM Jr. Protist homologs of the meiotic Spo11 gene and topoisomerase VI reveal an evolutionary history of gene duplication and lineage-specific loss. *Mol Biol Evol*. 2007, **24**, 2827–2841], Brochier-Armanet, Gribaldo and Forterre, manuscript in preparation). In the same vein, we have recently discovered that a universal protein of unknown function highly similar in Archaea and Splicea (Eukarya) is a new type of apurinic endonuclease (Hecker et al., 2007 [Hecker A, Leulliot N, Gadelle D, Graille M, Justome A, Dorlet P, Brochier C, Quevillon-Cheruel S, Le Cam E, van Tilbeurgh H, Forterre P. An archaeal orthologue of the universal protein Kae1 is an iron metalloprotein which exhibits atypical DNA-binding properties and apurinic-endonuclease activity in vitro. Nucleic Acids Res. 2007, 35, 6042–51]). We have also obtained *in silico *evidences for the existence of an ancient regulatory mechanism coupling DNA replication and translation in Archaea and Splicea (Berthon et al., 2008 [Berthon J, Cortez D, Forterre P. Genomic context analysis in Archaea suggests previously unrecognized links between DNA replication and translation. Genome Biol. 2008: 9, R71]). All these new findings point that the last common ancestor of Archaea and Splicea had a DNA genome. In the 2R/2V scenario, the independent RNA-DNA transition in bacteria and in a common ancestor to Archaea and Splicea could explain the more dramatic difference between the bacterial and the archaeal/spliceal versions of universal proteins, compared to the differences observed between the archaeal and the spliceal version. Note that in the 2R/2V scenario, the root of the universal tree of life should be located in the bacterial branch! So I could become finally a proponent of this idea that I have been previously fighting. This explains why I especially appreciate the comment of the authors on the compatibility between the bacterial rooting and a proto-eukaryotic like LUCA.

*[Author's response: We have taken good note of this breakthrough in the revised version {see ref 44}]*.

### Reviewers' report 3

Nicolas Galtier, Institut des Sciences de l'Evolution, Université Montpellier 2, France

This article reviews many aspects relevant to early stages of life on earth, focusing on the nature of LUCA, the last universal common ancestor. In summary, the authors claim/argue/suggest that:

- LUCA had introns and a splicing machinery

- LUCA had a nucleus

- eukaryotes emerged through phagocytosis, not fusion

- LUCA was not hyperthermophilic

- LUCA possessed a membran of the "sn1,2" (like in bacteria and eukaryotes), not "sn2,3" type

- LUCA was metabolically and genetically complex

- bacteria and archaea used to undergo reductive evolution

- most phylogenetic incongruences are explained by differential losses, not horizontal gene transfer

The overall picture is radically different from the prevailing view of a prokaryote-like LUCA with small-sized genome. Not all of these proposals are new, of course. This is more like an update over previous reviews by some of these authors. Recent literature makes them even more confident in their favourite "ancestral eukaryote" theory.

I found the paper fascinating with many respects:

- The topic is great. It is difficult to imagine a more fundamental (I mean, less applied) issue in biological sciences. Unraveling the origins of life is an appealing problem per se; nobody really cares, but everybody would like to know. I highly respect those of us who decide to devote their life to this useless, perhaps unreacheable goal.

- The bibliographic survey is thorough. Very useful. Nothing important is left apart. I learned much by reading this piece.

- Strong opinions are formulated (although we are talking about the biology of an organism which lived billions years ago). Quite stimulating.

- The form of the manuscript is unusual – a long, provocative review/opinion. I thank Biology Direct for letting such non-standard contributions be published.

I cannot comment on each and every point made by the authors, first because I am not enough qualified, and secondly because it would make the whole reading boring. I will focus on two general issues which I think most deserve to be debated.

1. Everything shared is ancestral?

Many of the developped arguments start from reporting shared cellular or genetic elements between extant species throughout the 3 domains of life, and conclude that these elements were already present in LUCA. Such elements include the nucleus (a nuclear-like structure has been discovered in Planctomyces, a bacterium), introns, (including self-splicing introns), and many genes found in several copies in extant genomes. The authors favour ancestral complexity followed by differential losses, rather than multiple inventions and/or horizontal gene transfers.

I think this view is highly respectable, but I must say I found the arguments (in favour of either hypothesis) not so strong. The fact that, for some gene phylogenies, several lineages trace back very early in evolution (earlier than the bacterial and archaeal ancestors) does not imply, I think, that these genes were duplicated in the genome of LUCA – Zhaxybayeva & Gogarten (2004 TIG), for instance, interpret this pattern in a very distinct way. Well, this depends much on what we call LUCA – see below comment 2.

*[Author's response: As you say, much depends on the assumptions made regarding the nature of LUCA. The paper by Zhaxybayeva and Gogarten that you refer to focuses on HGT and, from a qualitative point of view, we certainly agree with their main conclusion that "contributions of vertical inheritance and HGT are not the same across the tree of life". They make a passing allusion to paralogy as an alternative to HGT but they do not address our specific point, i.e deep ancestral paralogy as a predicted source of phylogenetic incongruencies. In elaborating our conception of LUCA, we have clearly been influenced by the "community" concept of Kandler and Woese (op cit) but also by a number of considerations regarding, the origin of the splicing machinery, the origin of thermophily, an urge to discuss the unwarranted assumption that everything evolves from simple to complex (cfr the ideas of SJ Gould and S. Kauffman, op cit), the no less unwarranted assumption that phylogenetic incongruencies are incontrovertible evidence of HGT at all levels of evolution and that the identification in a prokaryote of a gene homologous to a eukaryotic protein (such as actin or tubulin and many others) indicates that these prokaryotic versions should be regarded as ancestral rather than supposing their emergence in a protoeukaryotic ancestor. We have presented these considerations as a bundle of converging arguments. Certainly, much remains conjectural and some of our views clearly are provocative but we feel there is enough ground to consider them as an alternative to current thinking on the alleged "prokaryote to eukaryote" transition]*.

I note, furthermore, that some of the scenarios proposed by the authors require a fairly large number of independent losses. For instance, it is suggested that the bacterium which entered in endosymbiosis with a protoeukaryote, and eventually became the mitochondrion, could have not yet lost introns (inherited from LUCA). But this mitochondrial ancestor is well identified as an alpha-proteobacterium. Supposing that introns were present in the entire lineage linking LUCA to this organism would imply a very large number of independent losses of introns in bacteria (when not a single bacterial group kept them).

This remark also applies to the authors' proposal of ancestral nucleus and introns, subsequently lost by bacteria and archaea. By relying on the observation of nucleus-like structures in several prokaryotes (if I understand corectly), the authors's scenario implies a large number of losses of the nucleus. I note, however, that no "modern" eukaryote has ever lost the nucleus, although many of them have undergone reductive genome evolution (eg Microsporidians). If the phyletic distribution of nuclear-like structures is patchy, then assuming a homologous relationship between these forms appears little parsimonious, knowing that losing the nucleus is apparently not so common (at least for a eukaryote).

*[Author's response: Assuming extensive gene loss during emergence of certain lineages is indeed emphasized in our presentation; if the old idea of emergence of Bacteria and Archaea by "streamlining" of a more sophisticated ancestor is correct, it must have involved extensive gene loss indeed. Even among Eukarya, various instances of extensive evolutionary simplifications are known (see the flatworms, already mentioned, or the relative rarity of introns in eukaryotes with a prokaryotic life style, such as yeast). However we agree we have overstated the case regarding the fate of the nucleus by not discussing the alternative interpretation of independent occurrence of nuclei in different cell lines (the question of homology between the eukaryotic nucleus and that of Gemmata). This point has also been raised by Dr Poole and Dr Forterre and has been taken into account in the present version]*.

2. LUCA "community": what does it mean?

At several places in the manuscript, the authors evoke the LUCA "population" or "community". The two words have distinct definitions in the evolutionary literature: a population is a group of individuals from the same species, a community a group of ecologically related species. That these two terms are taken are synonymous is probably to be connected to the authors' (and others') conception of a weak or absent species structure at the time of LUCA: the 3 domains of life would have "crystallized" from a "communal population".

I must say I disagree with this view. I do not see any reason to believe that genetic evolution proceeded differently before vs. after the origins of modern bacteria, archaea and eukaryotes (which, by the way, did probably not occur simultaneously). The existence of species, i.e. entities such that genetic exchanges are much less frequent between than within, appears universal across current biodiversity, probably as a consequence of the existence of distinct, discrete ecological niches. Why would have it been different in the past?

*[Author's response: The main reason to think that the mode of genetic evolution was different in the era of LUCA {and before} from what it is now, is the assumption of genetic promiscuity between primitive cells presented originally by Kandler and Woese (op cit); however, to avoid possible confusions, we took some pain to emphasize that this idea in no way (at least in our mind) undermines the critical and unavoidable role of natural selection at all stages of biological evolution. In our answer to Dr Forterre we emphasize the contribution of S. Kauffman in this respect]*.

If we assume that "standard" species existed at the time of LUCA, then we must chose between the two terms, population or community. It seems to me that the high level of ancestral genetic, metabolic and ecologic diversity suggested by the manuscript (e.g., both mesophilic and thermophilic individuals) is not compatible with a single LUCA species – the word "community" should therefore be favoured. Now calling LUCA a "diversified community of species" appears to me equivalent to saying that the distribution of genetic and metabolic diversity across extant species was influenced by horizontal gene transfers between various ancestral species: several ancestors contributed to current collective gene pool. So perhaps the "ancestral redundancy" and "horizontal gene transfer" hypothesis discussed by the authors are not so contradictory.

*[Author's response: We have indeed used the words "population" and "community" as synonyms as far as LUCA was concerned but, in order to avoid possible confusions, we agree that the word "community" should prevail. On the other hand, our view certainly does not present LUCA as a single species nor as a diversified community of species, since, following Woese, we do not think standard (Darwinian) species existed at the time of LUCA. To use Kandler's words ", we see LUCA as a "multiphenotypic community" of cells, not endowed with the clear-cut differences maintained by genetic barriers that we see in modern lineages, because of the promiscuity assumed to have reigned at the time. To use your words, in such a community "several ancestors contributed to the current collective gene pool" so that "ancestral redundancy" and "horizontal gene transfer" were indeed not contradictory. However, if we do envisage that HTG was widespread at the time of LUCA, we would never consider it as ever having been "indiscriminate" (see Woese et al on aminoacyl-tRNA synthetases, Microbiol Mol Biol Rev 2000 64:202–236) since the outcome of gene exchanges, however widespread it may have been at this early time, always remained determined by natural selection]*.
